# A convective transport-enhanced multi-organoid device for therapeutic modeling of the liver-pancreas axis in obesity

**DOI:** 10.7150/thno.114415

**Published:** 2026-01-01

**Authors:** Jisu Kim, Jungho Bae, Baofang Cui, Seung-Woo Cho, Kisuk Yang

**Affiliations:** 1 Department of Bioengineering and Nano-Bioengineering, College of Life Sciences and Bioengineering, Incheon National University, Incheon, 22012, Republic of Korea.; 2 Department of Biotechnology, Yonsei University, Seoul, 03722, Republic of Korea.; 3 Center for Nanomedicine, Institute for Basic Science (IBS), Seoul, 03722, Republic of Korea.; 4 Cellartgen Inc., Seoul, 03722, Republic of Korea.; 5 Division of Bioengineering, College of Life Sciences and Bioengineering, Incheon National University, Incheon, 22012, Republic of Korea.; 6 Research Center for Bio Materials & Process Development, Incheon National University, Incheon, 22012, Republic of Korea.

**Keywords:** multi-organoid device, convective transport, organoids, metabolic dysfunction-associated steatotic liver disease, type 2 diabetes mellitus

## Abstract

**Rationale:** Obesity-associated metabolic diseases such as metabolic dysfunction-associated steatotic liver disease (MASLD) and type 2 diabetes mellitus (T2DM) are increasing rapidly, necessitating physiologically relevant *in vitro* models of the liver-pancreas axis. While recent multi-organoid systems have advanced inter-organ modeling, many systems still fall short of replicating the complex and directional metabolic interactions required to accurately reflect disease progression. This is partly due to inherent limitations such as reliance on passive diffusion for metabolic exchange and the use of shared or non-compartmentalized media, which restrict tissue-specific functions and fail to mimic *in vivo*-like physiological gradients.

**Methods:** To overcome these limitations, we developed a multi-organoid device (MOD) that incorporates convective flow and physically separates liver and pancreatic organoids in distinct media environments. To evaluate the effectiveness of this co-culture system, we assessed metabolic transport using FITC-dextran and examined pancreatic and liver organoid function by measuring insulin and albumin secretion respectively. The effectiveness of the MOD in modeling MASLD-induced T2DM was further validated through functional assays and transcriptomic analysis.

**Results:** The MOD successfully recapitulated key pathological features of MASLD-induced T2DM. Convective flow significantly enhanced directional transport of glucose and other metabolic molecules compared to passive diffusion, as validated by simulation and diffusion assays. Media separation preserved organoid function, increasing insulin and albumin secretion by 1.8- and 1.6-fold, respectively, compared with the non-separated group. Importantly, the device achieved rapid glucose regulation following glucose stimulation, with normoglycemia restored within 2 hours closely mimicking physiological glucose regulation not previously attainable in existing systems. Under MASLD conditions, the platform further revealed that liver-derived Fetuin-A was associated with β-cell apoptosis in pancreatic organoids.

**Conclusion:** This MOD effectively models the pathophysiological cascade linking MASLD and T2DM by integrating organ-specific environments, convective flow, and multi-organ crosstalk. It offers a robust and biologically relevant tool for mechanistic studies of metabolic diseases and provides a promising platform for preclinical drug screening and therapeutic development.

## Introduction

Obesity is a rapidly escalating global health concern, currently affecting over 650 million adults worldwide, and serves as a major risk factor for metabolic disorders such as metabolic dysfunction-associated steatotic liver disease (MASLD) and type 2 diabetes mellitus (T2DM) 1, 2. These diseases are closely interrelated and significantly contribute to morbidity and mortality through the disruption of glucose homeostasis and overall metabolic imbalance. The liver and pancreas play central roles in maintaining glucose homeostasis through tightly coordinated, bidirectional communication, primarily mediated by the hormones glucagon and insulin 3, 4. Dysregulation of this liver-pancreas axis, commonly observed in obesity-induced metabolic syndrome, is a key contributor to the onset and progression of T2DM.

To investigate the pathophysiology of T2DM and support the development of effective drug therapies, it is essential to establish *in vitro* models that accurately replicate inter-organ interactions and dynamic metabolic responses. However, disease modeling and drug discovery are often hindered by the substantial gap between *in vivo* physiology and conventional *in vitro* systems. In recent years, to address the limitations of traditional models, significant efforts have been made to develop three-dimensional (3D) tissue models, known as organoids, which more effectively reproduce the key structural and functional features of *in vivo* conditions 5. Organoids mimicking tissues such as the brain, liver, intestine, and pancreas have been developed by guiding stem cells to self-organize and differentiate into tissue-specific lineages 6-9. While these organoids show tremendous potential for both basic and applied research across fields, it is already apparent that single-tissue organoid models cannot recapitulate the complex interplay between tissues during the onset of a disease 10, 11.

To overcome the limitations of single-tissue organoids, substantial progress has been made in developing multi-organoid culture systems, including models of liver-pancreas interactions 10, 12-15. These systems integrate organoid technology with multi-organ platforms and offer an improved tissue microenvironment compared to traditional approaches like well plates by enabling inter-organ interactions and precise transmission of biomechanical and biochemical signals. However, these systems often fail to replicate essential disease-relevant features such as dynamic hormonal signaling and tissue-specific responses, limiting their accuracy in modeling complex metabolic disorders like T2DM. These limitations may stem from (1) slower metabolic transport rates compared to *in vivo* systems due to reliance on passive diffusion 16, which prevents the recreation of dynamic and physiologically relevant microenvironments that mimic blood flow-mediated metabolic exchange. The rapid transmission of hormonal signals particularly crucial in diseases like T2DM is often lacking in such systems. Systems utilizing convective transport have been explored to better mimic the rapid and directional flow of blood. Another limitation is (2) tissue degradation caused by the use of a single or mixed medium to culture tissues originating from different organs 17. To address this, it is important to design devices that allow each organ to be physically isolated yet still interact through biochemical signaling.

To address these limitations, we developed a multi-organoid culture system that incorporates convection-driven rapid molecular transport, provides separate culture chambers for each organoid, and connects them *via* a narrow channel referred to as the connecting channel using a semi-permeable polytetrafluoroethylene (PTFE) membrane. Our previous study confirmed that convective flow significantly enhances metabolic substrate transport compared to passive diffusion 16. We therefore hypothesized that applying convective transport in a multi-organoid setting could overcome current limitations in modeling obesity-induced metabolic dysfunction, particularly addressing challenges associated with slow metabolite exchange and limited inter-organ communication. To test this hypothesis, we modeled obesity-associated metabolic dysfunction in our system by inducing MASLD conditions in liver organoids through fatty acid exposure. The multi-organoid context allowed us to examine key pathological interactions between liver and pancreatic organoids, including hepatic lipid accumulation and subsequent impacts on pancreatic β-cell function and viability. Additionally, we employed transcriptomic analyses to uncover the molecular basis of inter-organ crosstalk under these obesity-mimicking conditions, highlighting differences that emerge uniquely during co-culture. Finally, we explored the potential of our platform as a physiologically relevant system suitable for therapeutic screening, particularly evaluating responses to anti-diabetic treatments such as metformin.

## Materials and Methods

### Design and fabrication of the Multi-Organoid Device (MOD)

The MOD was fabricated using the molding method 18 with polydimethylsiloxane (PDMS) (Dow Corning, MI, USA). The device consisted of three PDMS layers and a 1.0 μm pore size PTFE membrane (Merck Millipore, MA, USA) positioned between the top and middle layers. The top, middle, and bottom layers measured 6 mm, 1.5 mm, and 5 mm in height, respectively; all layers shared the same width (30 mm) and length (45 mm). The PDMS layers were fabricated by mixing a silicone elastomer base and curing agent at a 10:1 weight ratio, curing at 60 °C for 4 hours, and then irreversibly bonding the assembled layers using oxygen plasma treatment (Femto Science, Hwaseong, Republic of Korea). Pancreatic and liver organoids were placed in the pancreatic organoid chamber (POC) and liver organoid chamber (LOC), respectively, both located on the top PDMS layer above the PTFE membrane. The flow (600 μL/min) entered through the inlet, circulated through the connecting channel (CC) in the bottom layer, and exited through the outlet.

### Estimation of wall shear stress in the CC

To assess the mechanical microenvironment within the MOD, we estimated the wall shear stress experienced by organoids under perfusion. Shear stress was calculated using a simplified formula for flow in a rectangular channel, as previously described 19. In this equation, τ presents wall shear stress (dyne/cm²), μ is the dynamic viscosity of aqueous media at at 37 °C (~0.01 g/cm·s), Q is the flow rate (cm³/s), w and h are width and height (cm) of the channel, respectively:



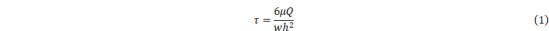



For the MOD, Q was set at 0.01 cm³/s (600 µL/min), with channel dimensions of 0.7 cm (w) and 0.3 cm (h). Substituting into the formula, the wall shear stress within the CC was calculated to be approximately 0.0095 dyne/cm².

### Generation of mouse tissue-derived liver organoids and pancreatic organoids

Mouse liver organoid (m-LO) and mouse pancreatic organoid (m-PO) generation were adopted from previously reported protocols, respectively 20-22. For organoid culture, 6-8-week-old C57BL/6 male mice (Nara Biotech, Seoul, Republic of Korea) were sacrificed, and liver and pancreas tissues were harvested and fragmented using scissors. All mouse experiments were approved by Institutional Animal Care and Use Committee (IACUC) of the Yonsei Laboratory Animal Research Center (YLARC) (permit number: IACUC-A-202403-1823-02). The liver and pancreas tissue pieces were incubated and rocked in digestion enzyme that contains 1:1 ratio (0.125 mg/mL each) of Collagenase (Sigma-Aldrich, MA, USA) and Dispase II (Sigma-Aldrich) in Dulbecco's modified Eagle medium (DMEM) high glucose (Thermo Fisher Scientific, MA, USA) medium supplemented with 1 % penicillin/streptomycin (Thermo Fisher Scientific) and 1 % fetal bovine serum (FBS) (Thermo Fisher Scientific) for 45 min at 37 °C. After enzymatic digestion, the supernatant of liver and pancreas was collected in 70 μm and 40 μm strainer, respectively and the remaining tissue fragments were pipetted vigorously with organoid basal medium (Advanced DMEM/F12 (Thermo Fisher Scientific) supplemented with 1 % penicillin-streptomycin (Thermo Fisher Scientific), 1 % HEPES (Thermo Fisher Scientific) and 1 % Glutamax (Thermo Fisher Scientific) to obtain a high yield of cells. Collected ductal cells, including stem cells, were centrifuged at 250 g, 4 °C for 5 min, and then resuspended in Ammonium-Chloride-Potassium (ACK) lysing buffer (Thermo Fisher Scientific) to remove red blood cells (RBCs). Centrifuged 40,000 ductal cells were seeded in 48-well plates encapsulated in 30 μL growth factor reduced Matrigel (Corning, NY, USA). Culture medium was added to the organoid-laden hydrogel constructs in each well of a 48-well plate.

### Mouse liver and pancreatic organoid culture

Mouse liver organoid isolation medium was composed of 30 % (v/v) Wnt3a-conditioned medium, 25 ng/mL mouse noggin (Thermo Fisher Scientific) and 10 μM Rho-associated coiled-coil containing protein kinase (ROCK) inhibitor (BioGems International, Inc., CA, USA) in the m-LO expansion medium (EM). m-LO EM contains organoid basal medium supplemented with 10 % (v/v) R-spondin1-conditioned medium, B27 (Thermo Fisher Scientific), N2 supplement (Thermo Fisher Scientific), 10 mM nicotinamide (Sigma-Aldrich), 1 mM N-acetylcysteine (Sigma-Aldrich), 50 ng/mL mouse epidermal growth factor (EGF) (Invitrogen, MA, USA), 100 ng/mL human fibroblast growth factor (FGF)10 (Thermo Fisher Scientific), 10 nM human gastrin Ⅰ (Sigma-Aldrich) and 50 ng/mL human hepatocyte growth factor (HGF) (Thermo Fisher Scientific). The mouse m-LO isolation medium was applied for the initial three days, followed by replacement with m-LO EM every two days and passaging every 5 to 7 days. For further differentiation of m-LO, the medium was changed into liver differentiation medium (DM). The mouse liver DM was composed of the basal medium supplemented with B27 supplement (with or without vitamin A), 1 mM N-acetylcysteine, 10 nM human gastrin I, 50 ng/mL recombinant mouse EGF, 100 ng/mL recombinant human FGF10, 50 nM A83-01 (Tocris) and 10 μM N-[N-(3, 5-difluorophenacetyl)-l-alanyl]-s-phenylglycine t-butyl ester (DAPT) (STEMCELL Technologies, Vancouver, Canada), and 3 μM dexamethasone (Sigma-Aldrich).

m-PO EM was composed of the basal medium supplemented with 10 % (v/v) R-spondin1-conditioned medium, B27 minus vitamin A (Thermo Fisher Scientific), 10 mM nicotinamide, 1 mM N-acetylcysteine, 50 ng/mL mouse EGF, 100 ng/mL human FGF10, 10 nM human gastrin I, and 25 ng/mL mouse Noggin. The medium was refreshed every 2 days, and passaging was executed after 7 days. To induce differentiation into pancreatic organoids after passaging on MOD, the medium was replaced with DM. The pancreatic organoid DM consists of m-PO EM minus R-spondin1-conditioned medium supplemented with 250 nM SANT-1 (Sigma-Aldrich), 0.1 μM retinoic acid (Sigma-Aldrich), 20 ng/mL betacellulin (Thermo Fisher Scientific), 1 μM XXI (Tocris), 10 μM Alk5 inhibitor (Alk5i) (Sigma-Aldrich) and 1 μM T3 (Sigma-Aldrich).

### Cell culture

A human hepatoma cell line (HepG2) (ATCC, Virginia, USA) was cultured in DMEM (Thermo Fisher Scientific) supplemented with 10 % FBS, 1 % penicillin-streptomycin. A Beta-TC-6 (MIN6, ATCC) was cultured in DMEM supplemented with 15 % FBS, 2 % penicillin-streptomycin. The cells were maintained in a humidified chamber containing 5 % CO_2_ at 37 ℃. In all experiments, HepG2 and MIN6 cell spheroids made using SpheroFilm^TM^ (INCYTO, Cheonan, Republic of Korea) were used and spheroid generation followed the protocol described in our previous study 23.

### Quantification of molecular transport and PTFE membrane diffusion

To evaluate the convective transport and diffusion characteristics within the MOD, fluorescein isothiocyanate (FITC)-dextran molecules were used as fluorescent tracers. To mimic insulin transport, 4 kDa FITC-dextran (10 µg/mL; Sigma-Aldrich), comparable in size to insulin (5.8 kDa), was introduced into the POC, and aliquots were collected from the LOC and CC. Conversely, to simulate Fetuin-A (FetA, 51-67 kDa) transport, 70 kDa FITC-dextran (10 µg/mL; Sigma-Aldrich) was added to the LOC, and aliquots were collected from the POC and CC.

In parallel, to assess media mixing and diffusion delay specifically through the PTFE membrane, 4 kDa and 70 kDa FITC-dextrans were separately added to one chamber in both separated device (SD) and non-separated device (NSD) configurations. Aliquots from the adjacent chamber were collected at defined intervals and analyzed for fluorescence using a plate reader (Varioskan LUX; Thermo Fisher Scientific).

### Measurement of membrane permeability

To assess glucose and size dependent membrane permeability, glucose and two different sizes of FITC-dextran (4 kDa and 70 kDa) were added to each chamber. According to the previously reported method 24, permeability was calculated with the below equation. In the equation, C_t_ is the concentration in the LOC or POC, C_0_ represents the initial concentration in the donor chamber, V denotes the volume of the medium within the CC, A is the membrane area in contact with cells, and Δt indicates the assay duration.



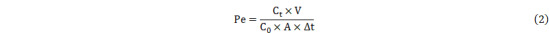



### Computational simulation

To predict molecular transport across the membrane, computational simulations were performed using COMSOL Multiphysics (COMSOL Inc., MA, USA). Simulations included glucose, insulin (modeled with 4 kDa FITC-dextran), and FetA (modeled with 70 kDa FITC-dextran). The flow generated by the peristaltic pump was simulated using the laminar flow interface, with the density and dynamic viscosity of the culture medium set to 1,030 kg/m^3^ and 0.0025 Pa·s, respectively 6, 25. The flow through the PTFE membrane was simulated using the Darcy's Law interface, with the porosity and permeability of the membrane set to 0.8 and 2.5 × 10^-14^ m^2^, respectively. The transport of glucose, 4 kDa FITC-dextran, and 70 kDa FITC-dextran within the MOD was simulated using the Transport of Diluted Species interface. The diffusion coefficients of glucose, 4 kDa FITC-dextran, and 70 kDa FITC-dextran in the culture medium were set to 9.58×10^-10^ m^2^/s, 1.35×10^-10^ m^2^/s, and 2.30×10^-11^ m^2^/s, respectively 26, 27. The initial concentration of glucose in the CC was 13 mM. The initial concentrations of 4 kDa FITC-dextran in the pancreas chamber and 70 kDa FITC-dextran in the LOC were 1 μg/mL. The initial concentration of all testing molecules in other regions of the MOD was set to 0 mM.

### Co-culture of liver and pancreatic organoids in MOD

To initiate co-culture of the m-LOs and m-POs within the MOD, organoids were encapsulated in 30 μL growth factor reduced Matrigel (Corning) and seeded into their respective chambers (LOC or POC). The device was first stabilized for 1 day in EM, then connected to peristaltic pump operated at a flow rate of 600 μL/min, followed by 3 days of culture in DM. Dulbecco's Phosphate Buffered Saline (DPBS) containing 5.5 mM glucose [with or without palmitate (PA)] flowed through the CC. The medium was refreshed daily.

### PA treatment

A 100 mM stock solution of palmitate (PA, Sigma-Aldrich) was prepared by dissolving PA in 50% ethanol (Thermo Fisher Scientific) at 70 ℃. Separately, a 10 % (w/v) bovine serum albumin (BSA, Sigma-Aldrich) solution was prepared in DPBS (Welgene, Gyeongsan, Republic of Korea) and kept at 37 ℃ in a water bath. PA was conjugated to 1 % fatty acid-free BSA and subsequently diluted to different working concentrations (0.2, 0.25, and 0.3 mM) prior to treatment.

### Quantitative real-time polymerase chain reaction (qRT-PCR)

The assay was conducted according to our previous protocol 11. Briefly, total RNA was extracted from each organoid using the Qiagen RNeasy Mini Kit (Qiagen, CA, USA), and complementary DNA (cDNA) synthesis was performed using a cDNA synthesis kit (TaKaRa, Shiga, Japan). Quantitative real-time PCR (qRT-PCR) analysis was performed using TaqMan® Fast Universal PCR Master Mix (Thermo Fisher Scientific) or Universal SYBR Green Fast qPCR Mix (Abclonal, MA, USA) on the StepOnePlus Real-Time PCR System (Applied Biosystems, MA, USA). Relative gene expression was quantified using the Comparative C_T_ (ΔΔC_T_) method, with glyceraldehyde 3-phosphate dehydrogenase (GAPDH). The following Taqman primers were used: hepatocyte nuclear factor 4 alpha (*Hnf4α*) (Mm01247712_m1), Keratin 18 (*Krt18*) (Mm01601704_g1), Keratin 19 (*Krt19*) (Mm00492980_m1), chromogranin A (*Chga*) (Mm00514341_m1), pancreatic duodenal homeobox 1 (*PDX1*) (Mm00435565_m1), NK6 homeobox 1 (*Nkx6.1*) (Mm00454961_m1), insulin 2 (*Ins2*) (Mm00731595_gH), and *Gapdh* (Mm99999915_g1). The primers for SYBR® Green used in this study were as follows: cytochrome P450 17A1 (*Cyp17a1*) (F:5′-AGCTCTGTGCTGAACTGGATCC-3', R:5′-AGACGGTGTTCGACTGAAGCCT-3′), albumin (*Alb*) (F:5′-CAGTGTTGTGCAGAGGCTGACA-3', R:5′-GGAGCACTTCATTCTCTGACGG-3′), and *Gapdh* (F:5′-CTTTGTCAAGCTCATTTCCTGG-3', R:5′- CTTGCTCAGTGTCCTTGC-3′).

### Immunocytochemistry staining

Immunocytochemistry staining was performed according to our previous research protocol 28. For immunostaining, organoids were released from growth factor reduced Matrigel (Corning) using Cell Recovery Solution (Corning), washed, and fixed in 4% paraformaldehyde for 30 minutes. They were then permeabilized with 0.2% Triton X-100 (Sigma-Aldrich) for 1 hour, followed by blocking with 4% BSA (MP Biomedicals, CA, USA) for 4 hours. Organoids were incubated with primary antibodies for 48 hours, including Rabbit anti-ALB (1:200, Abcam), Rabbit anti-PDX1 (1:200, Cell Signaling Technology, MA, USA), Mouse anti-KRT19 (1:200, Cell Signaling Technology), Mouse anti-CHGA (1:100, Santa Cruz Biotechnology, CA, USA), Mouse anti-AFP (1:100, Santa Cruz Biotechnology), Mouse anti-INS (1:100, Santa Cruz Biotechnology ), and Rabbit anti-Cleaved Caspase-3 (1:400, Cell Signaling Technology), followed by a 24-hour PBS wash. After washing, secondary antibodies were applied for an additional 24 hours: anti-mouse Alexa Fluor 488 (1:200, Thermo Fisher Scientific), and anti-rabbit Alexa Fluor 594 (1:200, Thermo Fisher Scientific). Nuclei were counterstained using 4′,6-diamidino-2-phenylindole (DAPI) (Vector Laboratories, CA, USA).

### Cell viability analysis

To assess cell viability, organoid culture samples were mixed at a 1:1 ratio with Trypan Blue (Thermo Fisher Scientific) to distinguish live and dead cells. A 10 μL aliquot of each mixture was then loaded onto a Countess™ cell counting chamber slide (Thermo Fisher Scientific) for automated cell counting. The slides were placed into an automated cell counter, and the number of live cells was recorded. In parallel, cell viability was also evaluated using the AlamarBlue assay (Thermo Fisher Scientific), performed at a 1:10 dilution. Then, cells were incubated with the reagent for 4-8 hours and analyzed using a plate reader (Thermo Fisher Scientific) by measuring absorbance at 570 nm, with normalization to the 600 nm reference wavelength. Additionally, live/dead staining was performed according to the manufacturer's instructions using the Live/Dead™ Viability Kit (Thermo Fisher Scientific).

### Oil red O staining

Oil Red O staining was performed using an Oil Red O Staining Kit (Thermo Fisher Scientific) according to the manufacturer's protocol. Briefly, cells were washed with PBS, fixed with 10 % formalin, and incubated for 30 minutes to 1 hour. After treatment with 60 % isopropanol for 5 minutes, the cells were stained with Oil Red O working solution. Subsequently, the stained lipids were extracted, and absorbance was measured at 492 nm using a plate reader for quantification.

### Analytical measurements of insulin and FetA secretion

Proteins from cells were collected at each time point and analyzed through the following Enzyme-Linked Immunosorbent Assay (ELISA) kit according to manufacturer's protocol: mouse fetuin-A/AHSG DuoSet, human fetuin-A/AHSG DuoSet, mouse serum albumin DuoSet, human serum albumin DuoSet (R&D Systems, MN, USA), and mouse insulin (Mercodia, Uppsala, Sweden). FetA samples were collected from the LOC, and insulin samples were obtained from the CC to reflect its secretion and transport across the membrane.

### FetA antibody-neutralization assay

To evaluate the involvement of FetA in β-cell apoptosis, a blocking experiment was performed using a mouse FetA antibody (R&D Systems, MN, USA). m-LO and m-PO were co-cultured in MOD and treated under the following conditions for 3 days: (1) Control (no treatment), (2) 0.3 mM palmitate, (3) 0.3 mM palmitate + 4 μg/mL anti-FetA antibody.

### Glucose-stimulated insulin secretion (GSIS) analysis

GSIS was performed according to the protocol described in the previous study 29. The MODs were prepared by embedding m-POs and m-LOs in reduced growth factor Matrigel (Corning) and culturing them in the POC and LOC, respectively. On day 1, organoids were incubated with EM and subsequently cultured under co-culture conditions (Co-culture and Co-culture+PA) in DM for 3 days. Subsequently, the m-POs were transferred from the device to a 48-well plate and starved with 300 µL of 2.8 mM glucose solution for 1 hour, followed by stimulation with 300 µL of 20 mM glucose solution for 1 hour. This glucose stimulation cycle was repeated three times. After each incubation, the solution from the POC was collected, and insulin concentrations were measured using a mouse insulin ELISA kit as described above.

### Glucose tolerance test (GTT) *in vitro*

GTT were conducted using the following experimental groups: Co-culture (control), Co-culture+PA, and Co-culture+PA+metformin. Glucose solution (13 mM) was injected into the injection port on day 4. Metformin (Sigma-Aldrich) was applied at 20 μM according to a previously reported protocol 30. The solution from the CC was collected at multiple time points, and glucose concentration was measured using the Amplex Red Glucose Assay (Thermo Fisher Scientific), while insulin concentration was also quantified using the mouse insulin ELISA Kit.

### RNA-sequencing analysis

Total RNA was extracted from cultured cells for RNA sequencing, as described in the qRT-PCR section. RNA quality was assessed by Agilent 4200 TapeStation System (Agilent Technologies, CA, USA), and RNA quantification was performed using Qubit (Thermo Fisher Scientific). For control and test RNAs, library construction was performed using QuantSeq 3'mRNASeq V2 Library Prep Kit FWD (Lexogen, Vienna, Austria) according to the manufacturer's instructions. In brief, each total RNA sample was prepared and an oligo-dT primer containing an Illumina compatible sequence at its 5' end was hybridized to the RNA and reverse transcription was performed. After degradation of the RNA template, second strand synthesis was initiated by a random primer containing an Illumina compatible linker sequence at its 5' end. The double stranded library was purified by using magnetic beads to remove all reaction components. The library was amplified to add the complete adapter sequences required for cluster generation. The finished library was purified from PCR components.

High-throughput sequencing was performed using single-end 100 bp reads on the NextSeq 2000 platform (Illumina Inc., CA, USA). Raw sequencing reads were preprocessed using fastp v0.23.1 to remove adapter sequences and low-quality reads 31. The filtered reads were then aligned to the reference genome using STAR v2.7.10b 32. The read counts were quantified using featureCounts v2.0.6 33. For differential gene expression analysis, edgeR package v3.42.4 was used 34, applying TMM-normalized log_2_(CPM + 1) transformation for data normalization.

### Statistical analysis

All data were first tested for normality using the Shapiro-Wilk test. As all datasets followed a normal distribution, parametric statistical tests were applied based on experimental design. One-way ANOVA was used to compare more than two groups under a single factor, such as different culture conditions. For experiments involving two independent variables, such as culture condition and time, two-way ANOVA was applied. When both between-subject factors (e.g., culture condition) and within-subject factors (e.g., repeated measures over time) were present, mixed ANOVA was employed to account for both sources of variability. For post hoc comparisons, Tukey's multiple comparison test was used for all pairwise group comparisons. Dunnett's test was applied when comparing multiple experimental groups directly to a control group.

Statistical significance thresholds were defined as *p* < 0.05, 0.01, 0.001, and 0.0001, and all data were presented as mean ± standard deviation (SD). Statistical analyses were performed using GraphPad Prism 8 (GraphPad Software, CA, USA), and all *in vitro* experiments included at least three independent biological replicates.

## Results

### Design of multi-organoid device (MOD) that recapitulates liver-pancreas axis

**Figure 1:**
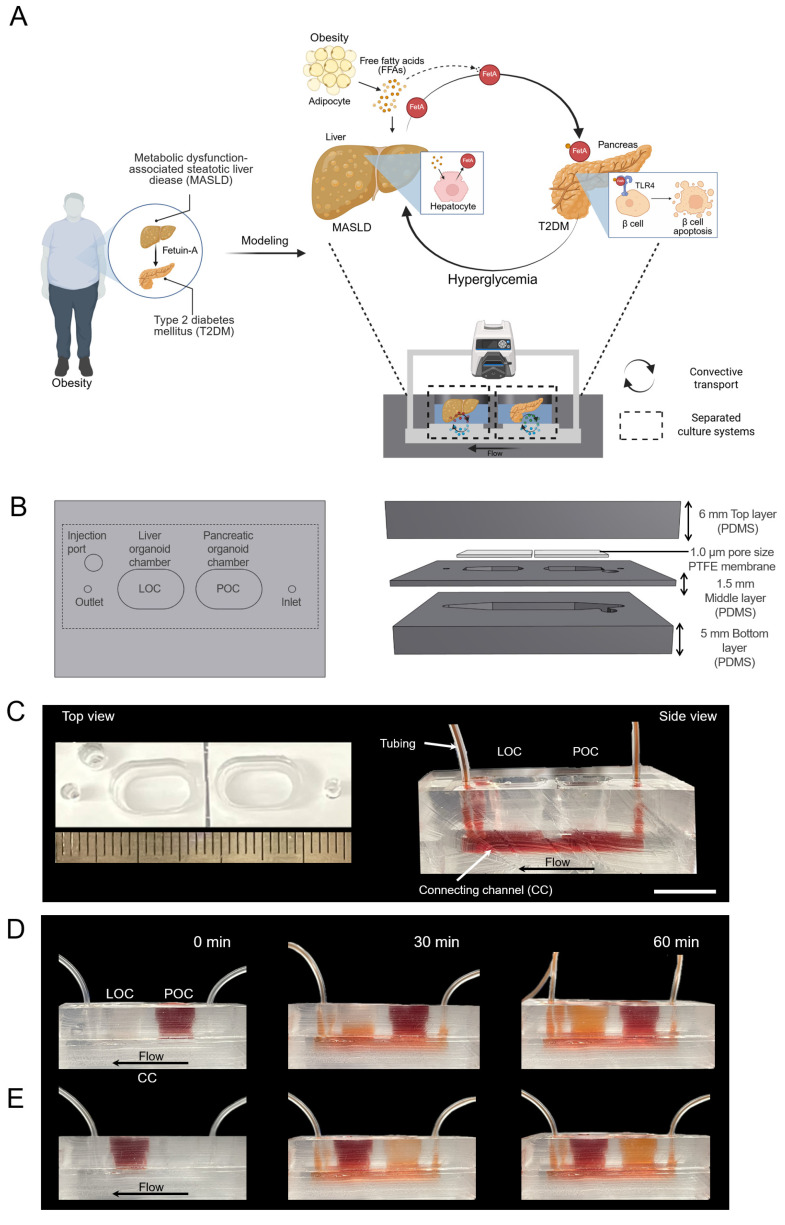
**Design of MOD enhanced with convective transport for modeling the liver-pancreas axis disease mechanism in obesity.** (A) Schematic illustration of MASLD and T2DM modeling *via* liver-pancreas organoid interactions in the MOD, highlighting inter-organ crosstalk relevant to obesity-associated pathophysiology. (B) Design of the MOD consisting of three PDMS layers with a 1.0 μm PTFE membrane. m-POs and m-LOs were cultured in each chamber of the top layer. (C) Photograph showing the top (left) and side view (right) of MOD. The flow enters through the inlet, passes through the connecting channel (CC) in the bottom layer, and exits through the outlet (Scale bar = 1 cm). (D, E) Directional molecular transport within the MOD using red dye (600 µL/min flow rate), convective movement from the pancreatic organoid chamber (POC) to the liver organoid chamber (LOC) (top) and from the LOC to the POC (bottom), visualizing selective inter-organ flow through the CC.

A convective MOD was developed to recapitulate obesity-driven metabolic interactions between liver and pancreatic organoids, modeling the progression of MASLD and T2DM (**Figure 1A**). Briefly, obesity is associated with the development of insulin resistance, particularly in adipose tissue, which leads to an increased release of free fatty acids (FFAs) into the bloodstream 35. These FFAs are then taken up by the liver, where they contribute to lipid accumulation, inflammation, and disruption of normal metabolic processes, ultimately influencing the onset of conditions like MASLD and T2DM 36. Approximately 27 % of these FFAs are PA 37, which at elevated levels can induce MASLD and increase FetA expression and secretion from liver 38. This FetA binds to FFA and FFA-FetA promotes β-cell apoptosis through the Toll-like receptor 4 (TLR4) signaling pathway and impairs insulin secretion, causing T2DM 39. Based on these insights, we assessed whether hallmark features of MASLD namely, lipid accumulation, lipotoxicity, and FetA secretion could be reproduced in the MOD by treating m-LOs with PA, thereby validating this model as a physiologically relevant *in vitro* platform for studying obesity-related metabolic disease 40, 41.

In this setup, metabolic interactions between m-LOs and m-POs occurred *via* a shared CC acting as a fluidic bridge. While the CC does not contain endothelial or vascular-like structures, it supports directional molecular exchange between compartments 42. To recapitulate physiological circulation dynamics, the MOD would need to transport signals at a similar speed to the human body. Human blood flows at a rate of about 5 L/min, and the average blood volume is 5 L 43, 44, generating a circulation time of about 1 minute. To achieve the same circulation time in our device, we used a peristaltic pump to maintain the circulation of media (600 µL) at a rate of 600 μL/min, ensuring that the entire media volume is circulated once per minute. Although a relatively high flow rate (600 µL/min) was applied to the device, the resulting shear stress calculated at the CC wall (~0.0095 dyne/cm²) was low. Considering the presence of the 1.0 μm PTFE membrane above the channel, the actual shear stress experienced by the organoids is expected to be even lower, indicating that the applied flow was sufficiently gentle to maintain organoid integrity. This shear level is also consistent with values reported in previous studies for preserving organoid viability 45.

As a simple and straightforward strategy for creating separate chambers for each organoid with its preferred media, the MOD was fabricated by replica molding method 18. The device, fabricated using PDMS, comprises a top layer, a PTFE membrane with a pore size of 1.0 μm in the middle, and a bottom layer (**Figure 1B-C**). Additionally, an injection port was added to enable targeted administration of high-concentration glucose and/or drugs. To support separated organ culture, the liver and pancreas organoid chambers were physically isolated to prevent direct media mixing. Instead, molecular exchange between the chambers occurred exclusively *via* CC, with directional flow from the inlet to the outlet. To visualize this selective transport, artificial blood (red-colored dye) was introduced into each organ chamber. When introduced into the POC, the dye gradually diffused into the adjacent LOC through the CC, creating a directed concentration gradient (**Figure 1D**). Conversely, when artificial blood was introduced into the LOC, it circulated through the system and gradually reached the POC, demonstrating directional and selective inter-organ communication within the device (**Figure 1E**).

### Establishment of the mouse adult stem cell-derived liver and pancreatic organoids

**Figure 2:**
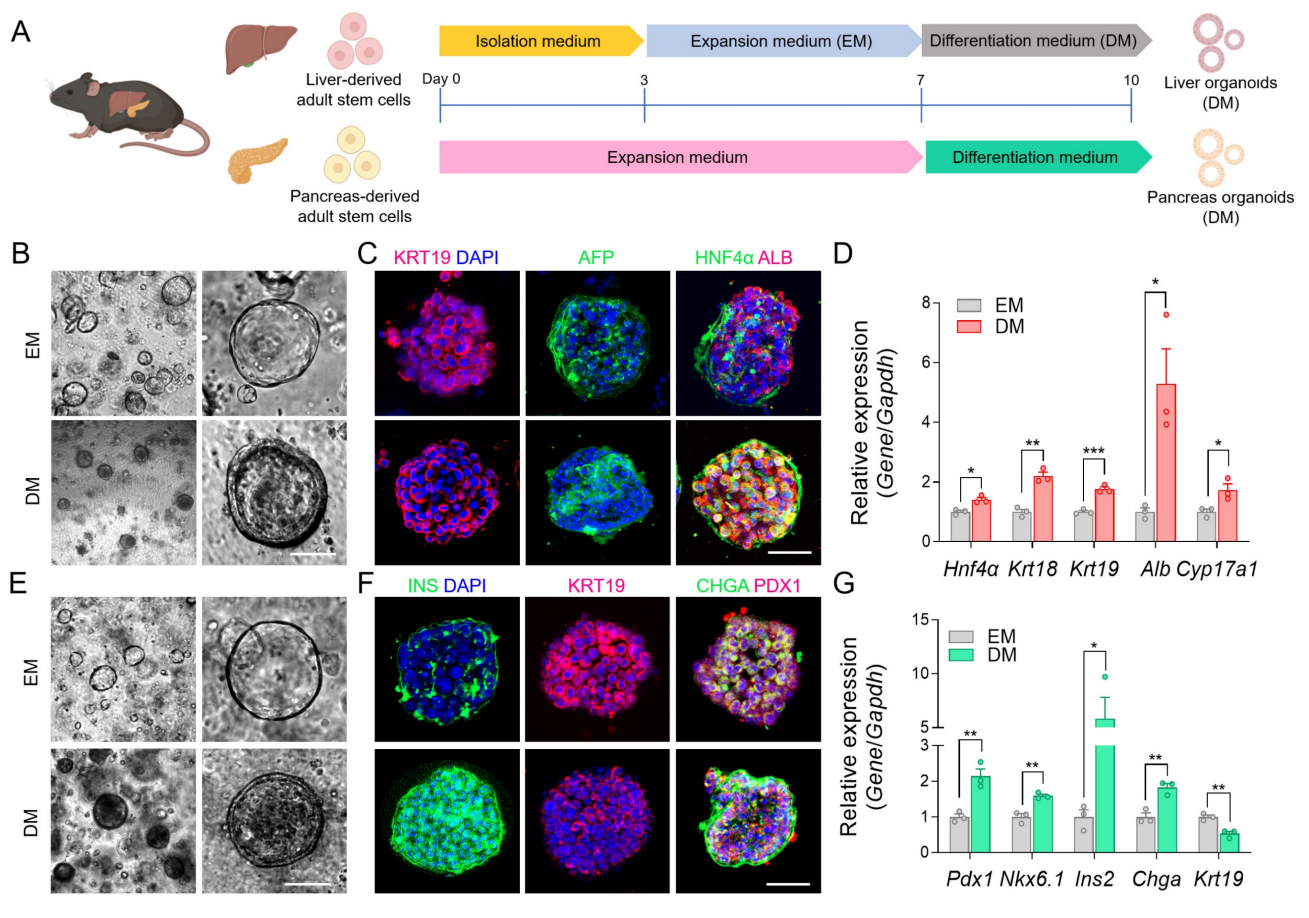
** Validation of organ-specific protein and gene expressions in liver and pancreatic organoids.** (A) Schematic illustration showing the step-by-step process for differentiating mouse tissue-derived stem cells into m-LOs and m-POs. (B) Bright-field images for differentiation-induced morphological changes to m-LOs (Scale bar = 50 μm). (C) Immunofluorescence staining of KRT19 (red), AFP (green), HNF4a (green), ALB (red) in m-LOs. DAPI was used to stain the nuclei (blue) (Scale bar = 50 μm). (D) Quantification of relative gene expression of *Hnf4α*, *Krt18*, *Krt19, Alb,* and* Cyp17a1* in m-LOs using qRT-PCR (n = 3, *p < 0.05, **p < 0.01, ***p < 0.001 vs. EM). (E) Bright-field images for differentiation-induced morphological changes to m-POs. (F) Immunofluorescence staining of INS (green), KRT19 (red), CHGA (green), PDX1 (red) in m-POs. (G) Quantification of relative gene expression of *Pdx1*, *Nkx6.1*, *Ins2*, *Chga,* and *Krt19* in m-POs by qRT-PCR (n = 3, *p < 0.05, **p < 0.01 vs. EM).

To mimic an *in vivo*-like environment, we established liver and pancreatic organoids derived from mouse liver and pancreatic stem cells 20, 22 that were differentiated to reach the proper state of liver function and pancreatic insulin secretion *via* DM cultures (**Figure 2A**). Differentiation induced notable morphological changes in liver organoids, including lumen thickening (**Figure 2B**). To evaluate the differentiation status, we performed immunofluorescent staining of KRT19, HNF4α, ALB, and AFP, which showed the expected staining patterns (**Figure 2C**). Here, HNF4α and ALB confirmed hepatocyte differentiation, AFP indicated residual immature hepatocyte features, and KRT19 reflected cholangiocyte lineage. We also confirmed these results using qRT-PCR, which showed increased gene expression levels for *Hnf4α*, *Krt18*, *Krt19, Alb,* and* Cyp17a1* following DM treatment (**Figure 2D**).

Next, we introduced an additional differentiation step using mature β cell differentiation factors, following previously published protocols, to promote β cell maturation in the pancreatic organoids 46. This differentiation led to notable morphological changes in pancreatic organoids, including a reduction in KRT19, which serves as a ductal lineage marker in the pancreas, while markers for pancreatic progenitors (PDX1), pan-endocrine cells (CHGA), and insulin production were upregulated 47, indicating the appropriate transition to functionally mature pancreatic organoids (**Figure 2E-F**). The qRT-PCR analysis also showed decreased expression of *Krt19*, while increased expression of *Pdx1*, *Nkx6.1*, *Chga*, and *Ins2* after differentiation (**Figure 2G**). These results confirmed the successful maturation of the organoids through DM, and the resulting differentiated liver and pancreatic organoids were subsequently integrated into the MOD.

### Increasing mass transfer through convective transport in the device

**Figure 3:**
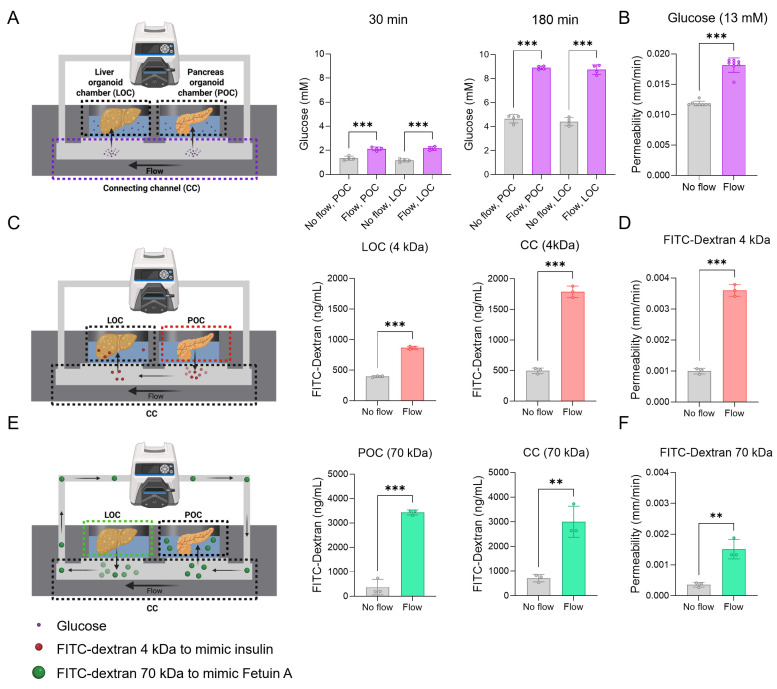
**Enhanced metabolic substrate transport by convective flow compared to passive diffusion.** (A) Schematic of glucose transport in the device, showing glucose with or without flow (600 μL/min), and the glucose concentration at 30 and 180 min (n = 4), along with (B) membrane permeability of glucose (n = 8, ***p < 0.001 vs. no flow). (C) Schematic of FITC-dextran (4 kDa) transport as an insulin-sized substitute, showing transport rates in devices and concentrations at 3 hours, and (D) membrane permeability (n = 3, ***p < 0.001 vs. no flow). (E) Schematic of FITC-dextran (70 kDa) transport as a FetA-sized substitute, with measured concentrations at 24 hours, and (F) membrane permeability (n = 3, **p < 0.01, ***p < 0.001 vs. no flow).

To address the limitations of simple diffusion in existing organoid devices, we employed convective flow to enhance the directional transport of metabolic substrates across organoid chambers. Key molecules involved in glucose homeostasis, insulin, and FetA were selected for evaluation in our system (**Figure 3A, C, E**). Glucose was introduced into the injection port at a concentration of 13 mM to mimic hyperglycemic conditions, while insulin and FetA were modeled by introducing 4 kDa and 70 kDa FITC-dextrans, respectively, into the POC and LOC. To determine the optimal PTFE membrane configuration for selective mass transfer while preventing cellular crossover 23, we tested membranes with 0.2 and 1.0 μm pores. The 1.0 μm membrane showed higher diffusion efficiency, and under flow conditions, it exhibited significantly enhanced diffusion for both 4 and 70 kDa FITC-dextrans (**Figure S1A**). Due to its ability to support efficient molecular transport, the 1.0 μm membrane was selected to preserve system integrity in subsequent experiments. Then, we confirmed that 4 and 70 kDa FITC-dextrans were selectively transmitted between the LOC and POC through the CC and across the PTFE membrane in a time-dependent manner. This inter-organ exchange was significantly enhanced in the presence of convective flow (**Figure S1B**).

As a first step, glucose transport from the CC to each organoid chamber was assessed under convective flow, which showed significant enhancement compared to static controls (**Figure 3A**). Similarly, 4 kDa FITC-dextran transport from the POC to the LOC increased under flow, with higher levels detected in both the CC and LOC compared to the no-flow group (No flow: 20.4 ± 3.1%; Flow: 58.4 ± 7.6% at 180 min) (**Figure 3C, Figure S1B**). Next, 70 kDa FITC-dextran introduced into the LOC, was transported through the circulation and delivered to the POC *via* the CC under flow conditions, resulting in significantly enhanced accumulation in both the CC and POC compared to the no-flow group (No flow: 15.9 ± 8.1%; Flow: 70.6 ± 12.4% at 24 h) (**Figure 3E, Figure S1B**). Quantitatively, convective transport increased the concentrations of glucose (at 180 min), 4 kDa (in the LOC at 3 hours), and 70 kDa FITC-dextrans (in the POC at 24 hours) by 1.9 ± 0.1-fold, 2.1 ± 0.1-fold, and 9.1 ± 0.3-fold, respectively, compared to the no flow group (**Figure 3A, C, E**). Measurement timepoints were chosen based on molecular size-dependent transport kinetics through the 1.0 μm pore membrane. Glucose and insulin, which are low molecular weight molecules, exhibit rapid inter-organ transport and physiological action within 2-3 hours; accordingly, their concentrations were assessed over a 3-hour period. In contrast, FetA (~70 kDa) has a substantially larger size and slower diffusivity across semipermeable membranes, with reported peak tissue accumulation occurring around 24 hours, justifying the extended observation window 48, 49. Additionally, we also showed an increase in membrane permeability for both glucose and FITC-dextran in the flow groups compared to no flow groups (**Figure 3B, D, F**).

To further validate our findings, we also performed COMSOL Multiphysics simulations for molecule-specific transport dynamics. Simulated profiles for glucose, 4 kDa FITC-dextran, and 70 kDa FITC-dextran closely matched the experimental transport measurements under flow conditions (**Figure S2**), supporting the accuracy of the model and design.

Together, these results demonstrate that convective transport achieved *via* flow increases metabolic substrate transport and suggest that the device partially mimics physiologically relevant circulation and metabolic signaling within the liver-pancreas axis, thereby supporting its potential as a platform for modeling inter-organ interactions relevant to obesity.

### Cell viability and function with separated culture media within MODs

Organoid dysfunction in existing systems has been attributed to the challenges of co-culturing different organoids in the same medium, which fails to meet the specific requirements of each organoid. Before applying our device to organoid culture, we first validated the impact of mixed-media conditions using a human hepatoma cell line (HepG2) and a mouse β cell line (MIN6). Viability of both cell types significantly decreased after 48 hours when cultured in either separate media or in a 1:1 mixture of both media, confirming the incompatibility of mixed-media conditions (**Figure S4A**). To assess the extent of inter-chamber media mixing, we performed a diffusion assay using FITC-dextran (4 kDa and 70 kDa). In the NSD, media components rapidly mixed between chambers, whereas in the SD, distinct fluorescence signals persisted for 24 hours, indicating delayed molecular diffusion and effective compartmentalization (**Figure S3**).

**Figure 4:**
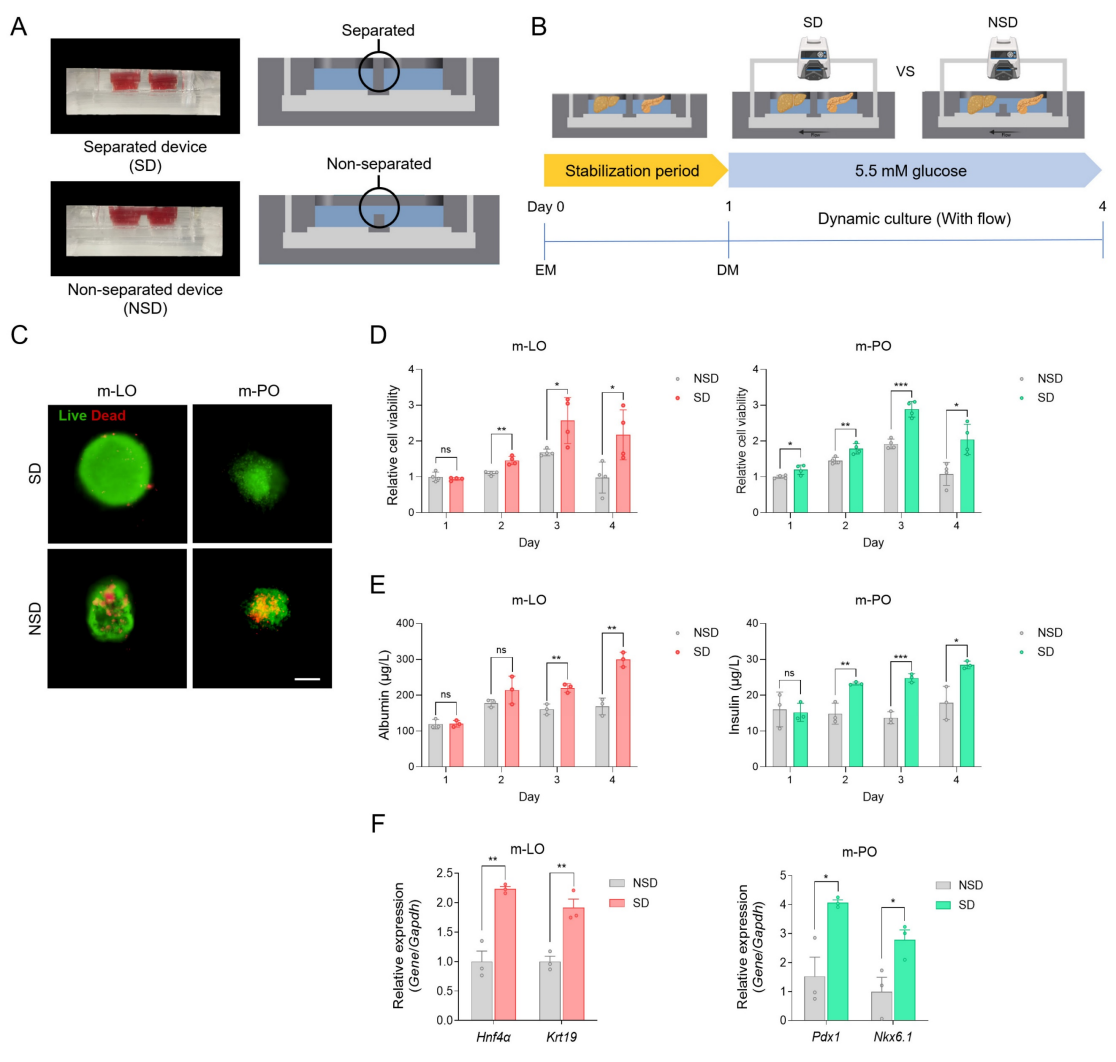
**Viability and functionality of organoids cultured in separated media within the MODs.** (A) Representative images and cross-sectional illustrations of separated device (SD) and non-separated device (NSD). (B) Schematic overview of the experimental workflow comparing SD and NSD under flow conditions. (C) Live/dead staining of m-LOs and m-POs after 4 days of culture in SD and NSD (Scale bar = 100 μm). (D) Cell viability of m-LOs and m-POs using AlamarBlue assay (n = 4, *p < 0.05, **p < 0.01, ***p < 0.001 vs. NSD). (E) ELISA assays of albumin secretion in m-LOs and insulin secretion in m-POs (n = 3, *p < 0.05, **p < 0.01, ***p < 0.001 vs. NSD). (F) Quantification of relative gene expression of *Hnf4α*, *Krt19* in m-LOs and *Pdx1*, *Nkx6.1* in m-POs using qRT-PCR (n = 3, *p < 0.05, **p < 0.01 vs. NSD).

After validating the effect of mixed-media conditions in cell lines, we extended our evaluation to organoid cultures within the MOD. We then configured the MOD as either an SD or an NSD to directly compare cell viability and function, including albumin secretion (m-LOs) and insulin secretion (β cells within m-POs) (**Figure 4A**). For co-culture, m-LOs and m-POs were seeded into the LOC and POC, respectively, stabilized in EM for 1 day, and then cultured in DM under flow conditions for 3 days to assess viability and function (**Figure 4B**). Time-course experiments were performed to evaluate cell viability and function over 4 days under both NSD and SD conditions. The results showed that SD conditions better maintained organoid viability over 4 days (**Figure 4C, D**), with viability sustained for up to 14 days (**Figure S5**), further supporting the advantage of media separation for long-term culture. In terms of functionality, albumin and insulin secretion levels were significantly higher under SD conditions, indicating improved hepatic and pancreatic organoid performance (**Figure 4E**). These findings were also observed in HepG2 and MIN6 cells using the same device setup (**Figure S4**). Furthermore, qRT-PCR analysis revealed significantly lower expression of hepatocyte (*Hnf4α*), cholangiocyte (*Krt19*), and pancreatic (*Pdx1 and Nkx6.1*) lineage markers under NSD conditions, indicating a loss of tissue identity in mixed media (**Figure 4F**). These findings suggest that the reduced function under non-separated conditions results from interference between organ-specific media components. Mechanistically, in NSD, betacellulin in pancreatic DM promotes β-cell differentiation at the expense of hepatocyte function in m-LOs 50, while DAPT in liver DM inhibits Notch signaling, leading to β-cell apoptosis in m-POs 51. However, the MOD (i.e. SD) preserved the cell viability and functionality of the organoids by delaying the mixing of their respective media through the utilization of a PTFE membrane. Thus, our results confirmed that organoid function improved in separated medium and that our device can reflect tissue-specific environments by providing separated tissue niches.

### Induction of MASLD-like conditions in the MOD

**Figure 5:**
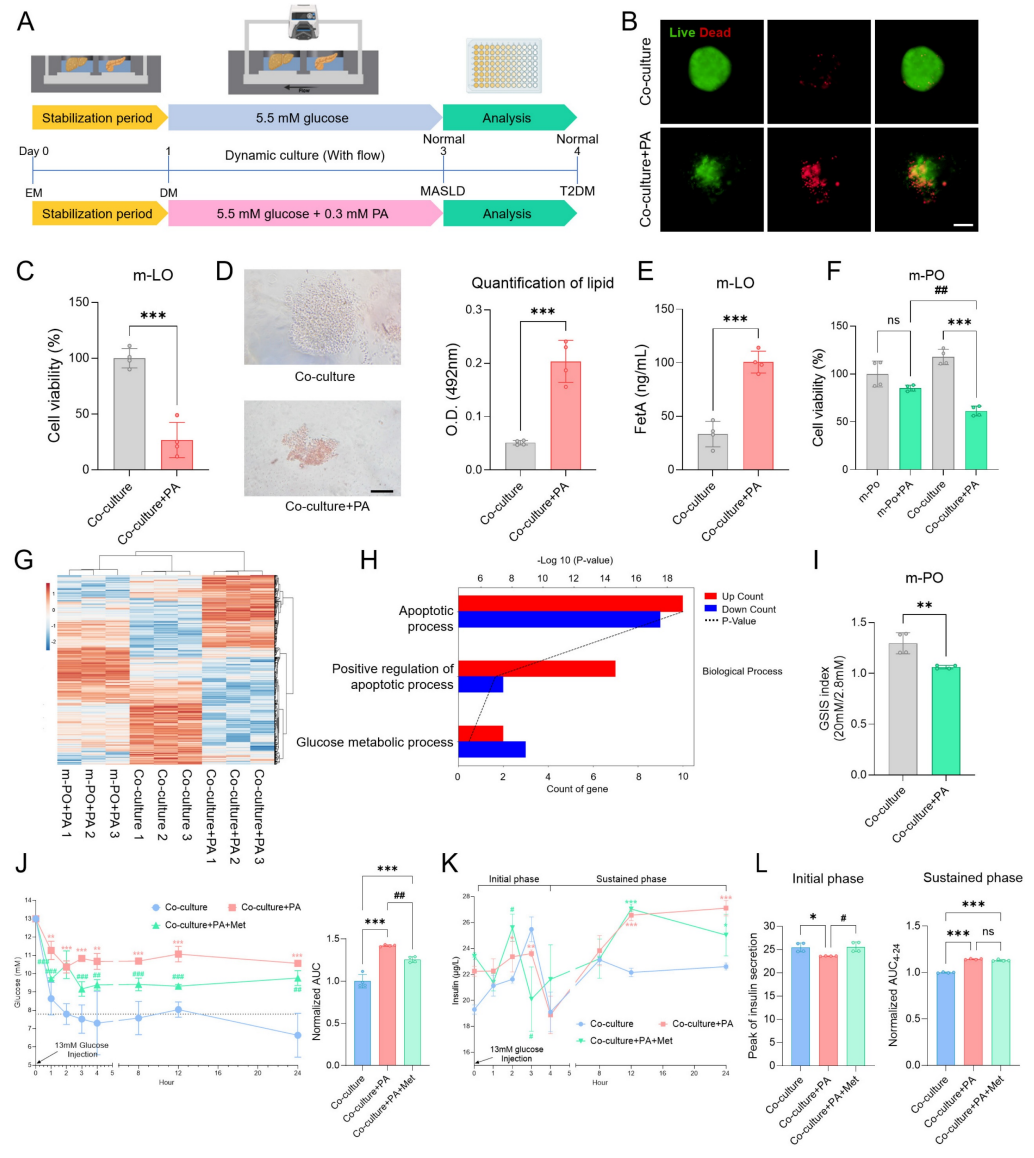
**Recapitulation of MASLD and T2DM pathophysiology and assessment of metformin in the MOD.** (A) Schematic overview of the experimental workflow. (B) Live/dead staining of m-LOs from Co-culture and Co-culture+PA conditions after 4 days in the device (Scale bar = 100 μm). (C) Cell viability of m-LOs in the device using alamarBlue assay (n = 4, ***p < 0.001 vs. Co-culture). (D) Oil Red O staining of m-LOs without or with PA treatment (Scale bar = 100 μm), and the quantification of lipid content (n = 4, ***p < 0.001 vs. Co-culture). (E) FetA secretion in m-LOs measured by ELISA (n = 4, ***p < 0.001 vs. Co-culture). (F) Cell viability of m-POs without or with PA treatment under monoculture and co-culture condition (m-PO, m-PO+PA, Co-culture, Co-culture+PA) using alamarBlue assay (n = 4, *p < 0.05, **p < 0.01, ***p < 0.001 vs. Co-culture, ^#^*p* < 0.05, vs. m-PO+PA). (G) Heatmap showing hierarchical clustering of gene expression profiles across three experimental groups: m-PO+PA, Co-culture and Co-culture+PA. (H) Gene Ontology (GO) enrichment analysis of differentially expressed genes (DEGs) between m-PO+PA and Co-culture+PA groups. (I) GSIS of m-POs (n = 4, **p < 0.01 vs. Co-culture). (J) Quantification of relative glucose levels during 24 hours following 13 mM glucose injection, along with the normalized area under the curve (AUC) for 24 hours (n = 4, **p < 0.01, ***p < 0.001 vs. Co-culture, ^##^*p* < 0.01, ^###^*p* < 0.001 vs. Co-culture+PA) (K) Insulin secretion by m-POs during GTT under different conditions during 24 hours after injection of 13 mM glucose (n = 4, *p < 0.05, **p < 0.01, ***p < 0.001 vs. Co-culture, ^#^*p* < 0.05, vs. Co-culture+PA). (L) Quantification of insulin peak secretion (μg/L) in initial phase and normalized AUC of insulin concentration in the sustained phase (n = 4, *p < 0.05, ***p < 0.001 vs. Co-culture, ^#^*p* < 0.05, vs. Co-culture+PA).

To first determine whether PA treatment induces MASLD in the MOD, we optimized the treatment conditions using HepG2 spheroids. Treatment with 0.3 mM PA induced MASLD-relevant phenotypes, including lipid accumulation, FetA secretion, and reduced cell viability, in line with previous reports 38, 40, 41 (**Figure S6**). Informed by these results, m-LOs cultured in the MOD were stabilized for 1 day in EM and then treated with 0.3 mM PA or left untreated (Co-culture) for 3 days in DM under flow conditions. Organoid analyses evaluating lipid accumulation, FetA secretion, and viability were then performed on day 4 (**Figure 5A**). After PA treatment, m-LOs exhibited disrupted morphology and decreased viability, both indicative of lipotoxicity (**Figure 5B-C**). Oil Red O staining revealed elevated lipid accumulation (**Figure 5D**), along with increased FetA secretion in the Co-culture+PA group (**Figure 5E**).

### MASLD-induced β cell apoptosis in the MOD

Next, we investigated whether MASLD-induced m-LOs contribute to T2DM pathophysiology by inducing β-cell apoptosis within m-POs. As an initial step, we assessed cell viability following treatment with 0.3 mM PA under two conditions: monoculture of m-POs and co-culture with m-LOs. In monoculture of m-POs, treatment with 0.3 mM PA did not significantly affect cell viability. However, in co-culture with m-LOs, the same treatment markedly reduced m-PO viability, suggesting the presence of liver-derived pro-apoptotic signaling (**Figure 5F**).

To further investigate whether this apoptotic effect originated specifically from MASLD-induced m-LOs, we performed RNA sequencing under the following three conditions: (1) m-POs treated with PA (m-PO+PA), (2) m-POs co-cultured with m-LOs under untreated conditions (Co-culture), and (3) m-POs co-cultured with PA-treated (MASLD-induced) m-LOs (Co-culture+PA). Hierarchical clustering revealed that the Co-culture+PA group exhibited a distinct gene expression profile, forming a separate cluster from the m-PO+PA and Co-culture groups (**Figure 5G**).

To clarify the molecular changes induced by co-culture with MASLD m-LOs, we conducted Gene Ontology (GO) enrichment analysis on differentially expressed genes. The analysis revealed a significant enrichment of apoptosis-related pathways in the Co-culture+PA group. Notably, both *apoptotic process* and *positive regulation of apoptotic process* were among the most significantly enriched GO terms, with the latter showing a larger proportion of upregulated genes (**Figure 5H**). These results suggest that co-culture with MASLD liver organoids induces β-cell apoptotic signaling.

### Recapitulation of MASLD-induced T2DM pathophysiology in the MOD

To confirm the induction of T2DM pathophysiology in the device, we evaluated whether the m-POs exhibited impaired insulin secretion, glucose intolerance, and hyperinsulinemia 52-54. To first confirm impaired insulin secretion, we performed the GSIS analysis, which showed significantly reduced insulin secretion in response to glucose stimulation in the Co-culture+PA group compared to the Co-culture (**Figure 5I**). Next, a GTT was conducted using the MOD by injecting 13 mM glucose into the injection port, circulating it through the CC to the POC, thereby mimicking postprandial blood glucose levels as described in a previous study 16. In the Co-culture+PA group, the glucose concentration initially decreased slightly but did not return to normal concentration, while in the Co-culture group, the glucose concentration decreased to the normoglycemic range within 2 hours (7.8 ± 0.5 mM) (**Figure 5J**). In contrast to the organoid co-cultures, where normoglycemia was restored within 2 hours, HepG2 and MIN6 cell co-cultures failed to achieve rapid glucose reduction. Instead, glucose levels declined only gradually, reaching the normoglycemic range (7.5 ± 0.1 mM) after 24 hours (**Figure S7**). This may be attributed to the lower insulin sensitivity of HepG2, a hepatoma cell line 55.

Overall, these GSIS and GTT *in vitro* analyses successfully modeled T2DM pathophysiology using the MOD. The selective transport of metabolic substrates, facilitated by convective flow, enabled glucose concentrations to quickly reach the normoglycemic range. In addition, these findings highlight that organoids more closely mimic physiological glucose dynamics compared to cell lines, suggesting greater suitability for modeling the liver-pancreas axis.

### Evaluation of metformin as a therapeutic intervention in the MOD

To evaluate whether the rapid decrease in glucose concentration was due to insulin secretion from β-cells within the m-POs, insulin measurement experiments were conducted. At the same time, to assess the potential of the MOD as a drug screening platform, we applied metformin, a widely used drug for T2DM, to evaluate its effects within our system. Metformin works by inhibiting the gluconeogenesis pathway in the liver and activating the AMP-activated protein kinase (AMPK) pathway, which promotes glucose uptake into cells 56. Then, it upregulates pancreatic aquaporin-7 expression, stimulating insulin secretion of the β cells 57. These mechanisms collectively reduce blood glucose levels in T2DM, making metformin an ideal candidate to test within our device.

To simultaneously evaluate hyperinsulinemia, a characteristic feature of T2DM, and assess the impact of metformin treatment on insulin secretion, we measured insulin levels from m-POs during a GTT under three conditions: Co-culture (control), Co-culture+PA, and Co-culture+PA+Met (Metformin treated) groups. Results from the GTT first confirmed the efficacy of metformin treatment, as evidenced by a significant reduction in glucose concentration within 1 hour compared to the Co-culture+PA group (**Figure 5J**). During the initial response phase (0-4 h), both the control and Co-culture+PA+Met groups exhibited a sharp increase in insulin secretion, which peaked and subsequently declined (**Figure 5K**). In contrast, the Co-culture+PA group displayed only a slight increase in insulin levels compared to the Co-culture group. At later time points (12-24 hour), insulin concentrations in the Co-culture group stabilized near baseline levels (1 h: 21.1 ± 0.8 µg/L; 12 h: 22.2 ± 0.3 µg/L; 24 h: 22.6 ± 0.1 µg/L), suggesting a return to homeostasis. In contrast, the Co-culture+PA group exhibited a sustained increase in insulin secretion (1 h: 22.3 ± 0.9 µg/L; 12 h: 26.6 ± 0.4 µg/L; 24 h: 27.1 ± 0.5 µg/L), indicative of persistent β-cell activation and a potential state of hyperinsulinemia. Notably, insulin levels in the Co-culture+PA+Met group remained comparable to those in the Co-culture+PA group, particularly during the sustained phase (4-24 hour), indicating that metformin did not fully normalize insulin secretion in this system (**Figure 5L**). While metformin is known to act on multiple tissues, including liver, muscle, and adipose tissue 58, our device focuses specifically on liver-pancreas interactions.

Furthermore, insulin secretion from β cells within the m-POs exhibited a biphasic pattern, consistent with typical *in vivo* observations 59. In the initial phase (0-4 hour), insulin secretion increased rapidly in response to glucose. Subsequently, after decreased insulin secretion, the sustained phase of gradual insulin secretion persisted 60. Overall, these data show that key characteristics of T2DM were observed in the MOD, successfully replicating the MASLD-induced T2DM model and demonstrating its potential as a drug screening platform.

### Confirming the mechanism of FetA-Mediated β-Cell Dysfunction in a MOD

**Figure 6:**
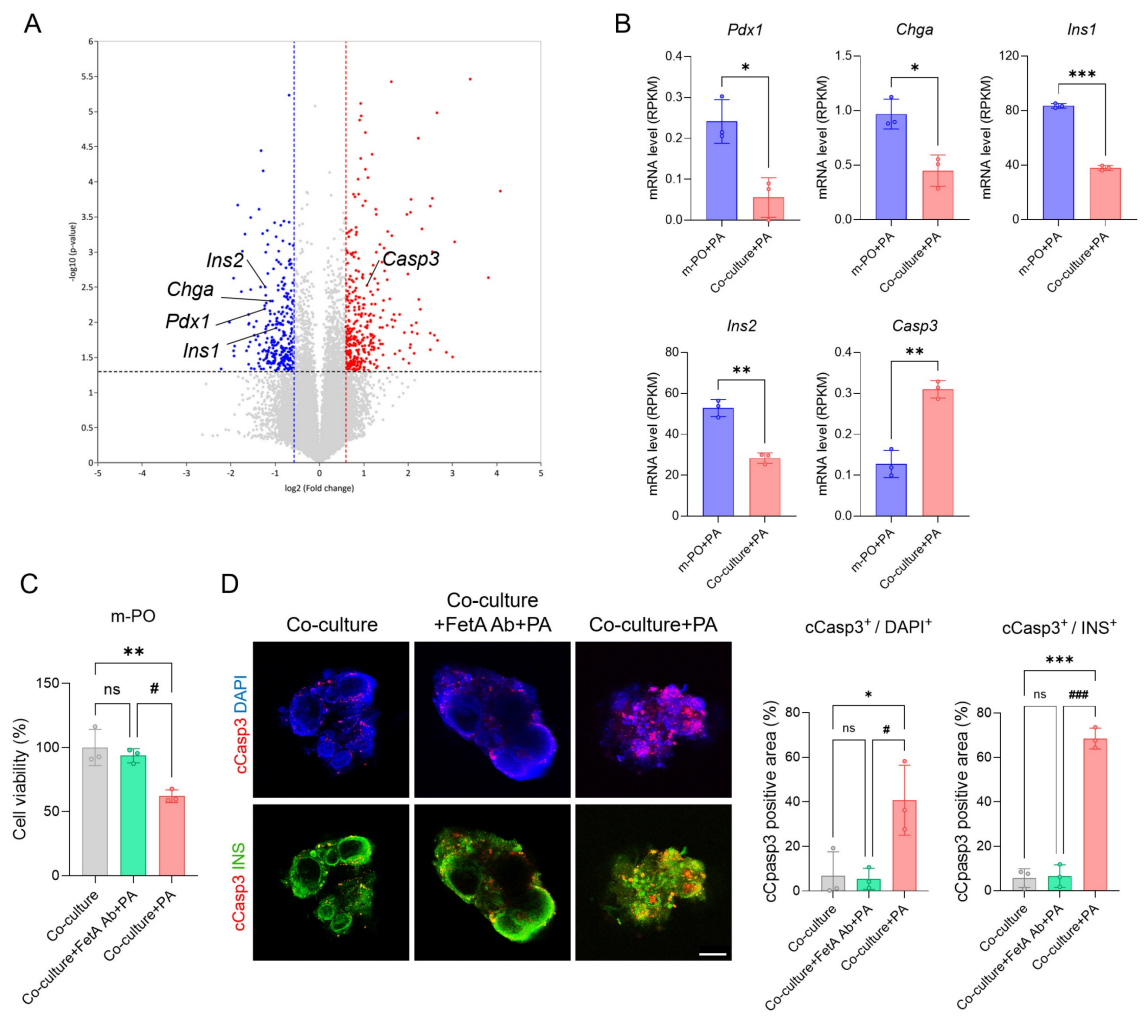
** Mechanism of FetA related inter-organ signaling associated with β-cell apoptosis in m-POs.** (A) Volcano plot showing significantly differentially expressed genes between m-PO+PA and Co-culture+PA group. (B) Expression levels of pancreatic β-cell markers (*Pdx1*, *Chga, Ins1*,* Ins2*) and apoptosis gene (*Caps3*) from RNA-seq analysis (n = 3, *p < 0.05, **p < 0.01 ***p < 0.001 vs. m-PO+PA). (C) Cell viability of m-POs under different conditions (Co-culture, Co-culture+FetA Ab+PA, Co-culture+PA), assessed using the alamarBlue assay (n = 3, **p < 0.01 vs. Co-culture, ^#^*p* < 0.05 vs. Co-culture+FetA Ab+PA). (D) Immunofluorescence staining of cCasp3 (red), INS (green) in m-POs and quantification of DAPI^+^ and cCasp3^+^ area per organoid and quantification of INS^+^ and cCasp3^+^ area per organoid (n = 3, *p < 0.05, ***p < 0.001 vs. Co-culture, ^#^*p* < 0.05, ^###^*p* < 0.001 vs. Co-culture+FetA Ab+PA). DAPI was used to stain the nuclei (blue) (Scale bar = 200 μm).

To further validate disease mechanisms within the MOD and demonstrate the importance of multi-organ interactions in modeling metabolic diseases, we analyzed transcriptomic changes at the molecular level. Consistent with transcriptomic data, volcano plot analysis revealed significant dysregulation of genes associated with β-cell identity and apoptosis (**Figure 6A**). Key β-cell markers including *Pdx1*, *Chga*, *Ins1*, and *Ins2* were downregulated, whereas the apoptosis-related gene, *Casp3* was notably upregulated in the Co-culture+PA condition compared to Co-culture group (control). These transcriptomic alterations were further supported by Reads Per Kilobase Million (RPKM)-based quantification, a normalization method used in RNA sequencing (**Figure 6B**), indicating that inter-organ signaling from MASLD liver organoids contributes to β-cell apoptosis within the MOD.

To strengthen the mechanistic link between FetA and β-cell apoptosis, we also conducted an antibody-neutralization experiment using a FetA-specific neutralizing antibody. In this experiment, cell viability significantly decreased in the Co-culture+PA group, whereas co-treatment with PA and the FetA antibody (Co-culture+FetA Ab+PA) prevented this reduction (**Figure 6C**). These findings confirm that FetA secreted from MASLD-induced m-LOs contributes to β-cell apoptosis, consistent with previous studies 39. To confirm that reduced viability reflected β-cell apoptosis, we performed immunofluorescence staining of m-POs using cleaved caspase-3 (cCasp3) and insulin (INS) antibodies, along with DAPI nuclear counterstaining. Quantification of cCasp3^+^/DAPI^+^ and cCasp3^+^/INS^+^ areas per organoid revealed significantly increased apoptosis in the PA-treated group compared to both the control and FetA Ab+PA treated groups. Notably, the cCasp3^+^/INS^+^ ratio reached 68.6 ± 4.6% in the PA-treated group, indicating widespread apoptosis among β-cells (**Figure 5D**).

Together, these results confirm that FetA secreted by MASLD-induced liver organoids plays a central role in mediating β-cell apoptosis within the MOD, contributing to T2DM-like pathophysiology. This supports the validity of our device as a disease-relevant platform and highlights the mechanistic importance of inter-organ signaling in metabolic disease modeling.

## Discussion

Glucose homeostasis is maintained through tightly regulated interactions between multiple organs, with the liver and pancreas playing central roles in this metabolic axis 4. Disruption of this inter-organ communication commonly induced by obesity can lead to metabolic disorders such as metabolic MASLD and T2DM. Although multi-organoid systems have advanced significantly, conventional co-culture platforms still struggle to reproduce physiologically dynamic metabolic responses. These limitations reflect the lack of active mass transport, leading to inefficient molecular exchange, and the dependence on shared, non-specific culture media, which undermines the preservation of organ-specific microenvironments 10, 15. Such limitations hinder accurate modeling of rapid glucose regulation and disease progression, ultimately reducing the utility of these systems for therapeutic drug discovery.

To address these challenges, a MOD was developed to emulate circulatory dynamics through convective transport, enabling fast and efficient metabolite exchange. By integrating a peristaltic pump, the system recreates the directionality and velocity of human blood flow 42-44, thereby enhancing the physiological fidelity of glucose and hormone dynamics. In addition, the MOD was designed to maintain organ-specific culture media in physically separated chambers for liver and pancreatic organoids, permitting selective molecular exchange through a semi-permeable PTFE membrane. This design resolves two major limitations of previous platforms, reducing their dependence on passive diffusion and preventing tissue dysfunction induced by mixed-media conditions. Using this platform, rapid glucose regulation was recapitulated *in vitro*, as evidenced by the return to normoglycemia within two hours following high-glucose infusion, a result not previously achieved in existing models 10, 14, 15. Together, these features highlight the strength of the MOD in supporting physiologically relevant inter-organ interactions.

While this configuration effectively minimizes cross-compartmental interference, it is important to note that small, bioactive molecules may still traverse the membrane over extended periods due to their low molecular weight. Although not all such molecular exchanges were quantitatively assessed in this study, the spatial separation of the two organoid chambers, combined with directional flow through the CC, enabled selective molecular transport, while the semi-permeable membrane contributed to the temporal attenuation of small-molecule diffusion. Together, these features contributed to enhanced viability and function of each organoid type within the MOD, ultimately supporting faster and more accurate metabolic responses.

With the ability of the MOD to preserve organ-specific functions and support accurate metabolic responses, the platform is well suited for investigating pathological inter-organ signaling. Consistent with this capability, exposure to PA led liver organoids to exhibit characteristic MASLD phenotypes, which in turn affected pancreatic organoids through FetA-mediated signaling 38, 40, 41. This inter-organ communication resulted in β-cell apoptosis and impaired insulin secretion, highlighting the pathological relevance of FetA in obesity-associated T2DM 3, 39, 61. Notably, blocking FetA signaling restored β-cell viability, supporting its causal involvement. Transcriptomic analyses further corroborated these findings, revealing that co-culture with MASLD liver organoids induced gene expression changes consistent with β-cell dysfunction. Collectively, these results underscore the importance of liver-derived signals in mediating β-cell damage and demonstrate that these mechanisms can be recapitulated within the MOD, supporting its potential as a relevant *in vitro* model for studying inter-organ metabolic disease.

Having established the utility of the MOD for modeling inter-organ disease mechanisms, the platform may also have potential for future drug screening applications. To ensure reliability in this context, it is important to address compound absorption associated with PDMS and to improve scalability for high-throughput use. A well-recognized limitation of PDMS is its tendency to absorb small hydrophobic molecules, which can interfere with drug concentration and assay consistency. In this study, metformin was selected as a hydrophilic compound with low PDMS absorption, and exposure duration was limited to minimize potential interference. While these conditions were sufficient for the present application, future iterations of the platform may incorporate surface modifications or alternative materials to improve compatibility with a broader range of drug candidates. In addition, modular configurations of the MOD and parallelized culture systems are being considered to enhance scalability and enable higher-throughput screening.

Beyond drug validation, the platform also holds potential for modeling inter-organ communication across other physiological systems. In the future, it could be applied to study organ interactions beyond the liver-pancreas axis, where efficient transport of signaling molecules and nutrients is essential for maintaining crosstalk (e.g., brain-gut or liver-gut axis). Although mouse-derived organoids were used in this study, employing human or patient-derived organoids would further enhance translational relevance. Moreover, incorporating vascular structures, immune components, and insulin clearance mechanisms will be important for improving physiological accuracy and more faithfully capturing complex systemic interactions.

## Conclusion

In summary, this study developed a MOD that addresses key limitations of existing platforms, particularly their dependence on passive diffusion and the mixed-media conditions that compromise tissue function. By introducing convective flow and maintaining separated culture environments, the system enabled efficient and directional transport of metabolic substrates such as glucose, insulin, and FetA, while preserving liver and pancreatic organoid function. The MOD captured key features of MASLD-induced T2DM, including lipid accumulation, β-cell dysfunction, and inter-organ crosstalk, and further allowed evaluation of therapeutic response, as demonstrated with metformin treatment. Together, these results highlight the value of the MOD as a physiologically relevant *in vitro* model for studying metabolic disease mechanisms and supporting drug validation.

## Supplementary Material

Supplementary figures.

## Abbreviations

MASLD: metabolic dysfunction-associated steatotic liver disease
T2DM: type 2 diabetes mellitus
MOD: multi-organoid device
3D: three-dimensional
PTFE: polytetrafluoroethylene
PDMS: polydimethylsiloxane
POC: pancreatic organoid chamber
LOC: liver organoid chamber
CC: connecting channel
m-LO: mouse liver organoid
m-PO: mouse pancreatic organoid
DMEM: Dulbecco's modified Eagle medium
FBS: fetal bovine serum
ACK: Ammonium-Chloride-Potassium
RBCs: red blood cells
ROCK: Rho-associated coiled-coil containing protein kinase
EM: expansion medium
EGF: epidermal growth factor
FGF: fibroblast growth factor
HGF: hepatocyte growth factor
DAPT: N-[N-(3, 5-difluorophenacetyl)-l-alanyl]-s-phenylglycine t-butyl ester
Alk5i: Alk5 inhibitor
FITC: fluorescein isothiocyanate
SD: separated device
NSD: non-separated device
DPBS: Dulbecco's Phosphate Buffered Saline
PA: palmitate
BSA: bovine serum albumin
cDNA: complementary DNA
qRT-PCR: Quantitative real-time Polymerase Chain Reaction
GAPDH: glyceraldehyde 3-phosphate dehydrogenase
Hnf4α: hepatocyte nuclear factor 4 alpha
Krt18: Keratin 18
Krt19: Keratin 19
Chga: chromogranin A
PDX1: pancreatic duodenal homeobox 1
Nkx6.1: NK6 homeobox 1
Ins2: insulin 2
Cyp17a1: cytochrome P450 17A1
Alb: albumin
cCasp3: cleaved caspase-3
DAPI: 4′,6-diamidino-2-phenylindole
ELISA: Enzyme-Linked Immunosorbent Assay
GSIS: Glucose-stimulated insulin secretion
GTT: Glucose tolerance test
FFAs: free fatty acids
TLR4: Toll-like receptor 4
AMPK: AMP-activated protein kinase
Met: metformin
RPKM: Reads Per Kilobase Million
