# Supplementary material for: A convective transport-enhanced multi-organoid device for therapeutic modeling of the liver-pancreas axis in obesity
Source: Theranostics. 2026 Jan 1;16(1):220–38. doi: 10.7150/thno.114415 (PMC12665103; doi:10.7150/thno.114415)
Supplement: Supplementary file 1 — Supplementary figures. [file thnov16p0220s1.pdf]

Supporting Information

A convective transport–enhanced multi–organoid device for therapeutic modeling of the liver–pancreas axis in obesity

Jisu Kim[1+], Jungho Bae[2+], Baofang Cui[2], Seung-Woo Cho[2,3,4*], Kisuk Yang[1,5,6*]

[1]Department of Bioengineering and Nano-Bioengineering, College of Life Sciences and Bioengineering, Incheon National University, Incheon, 22012, Republic of Korea. [2]Department of Biotechnology, Yonsei University, Seoul, 03722, Republic of Korea. [3]Center for Nanomedicine, Institute for Basic Science (IBS), Seoul, 03722, Republic of Korea. [4]Cellartgen Inc., Seoul, 03722 Republic of Korea. [5]Division of Bioengineering, College of Life Sciences and Bioengineering, Incheon National University, Incheon, 22012, Republic of Korea. [6]Research Center for Bio Materials & Process Development, Incheon National University, Incheon, 22012, Republic of Korea.

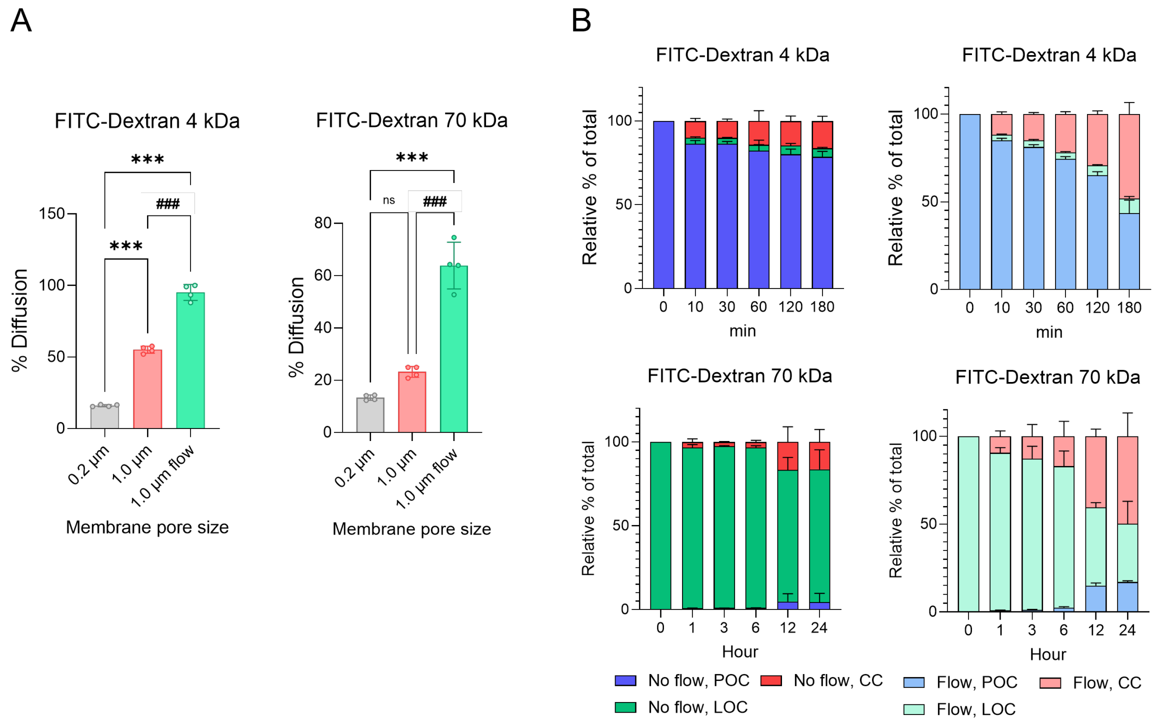

Figure S1. Mass transfer evaluation through PTFE membranes in convection flow-based multi-organoid device. (A) FITC-dextran (4 and 70 kDa) diffusion rate across 0.2 μm and 1.0 μm pore size PTFE membrane for 24 hours (n = 4, ***$p < 0.001$ vs. 0.2 μm, $^{###}p < 0.001$ vs. 1.0 μm). (B) Evaluation of substance mass transfer efficiency between separated chambers (pancreatic organoid chamber, POC and liver organoid chamber, LOC) and the connecting channel (CC) through convection flow using a 1.0 μm PTFE (n = 4).

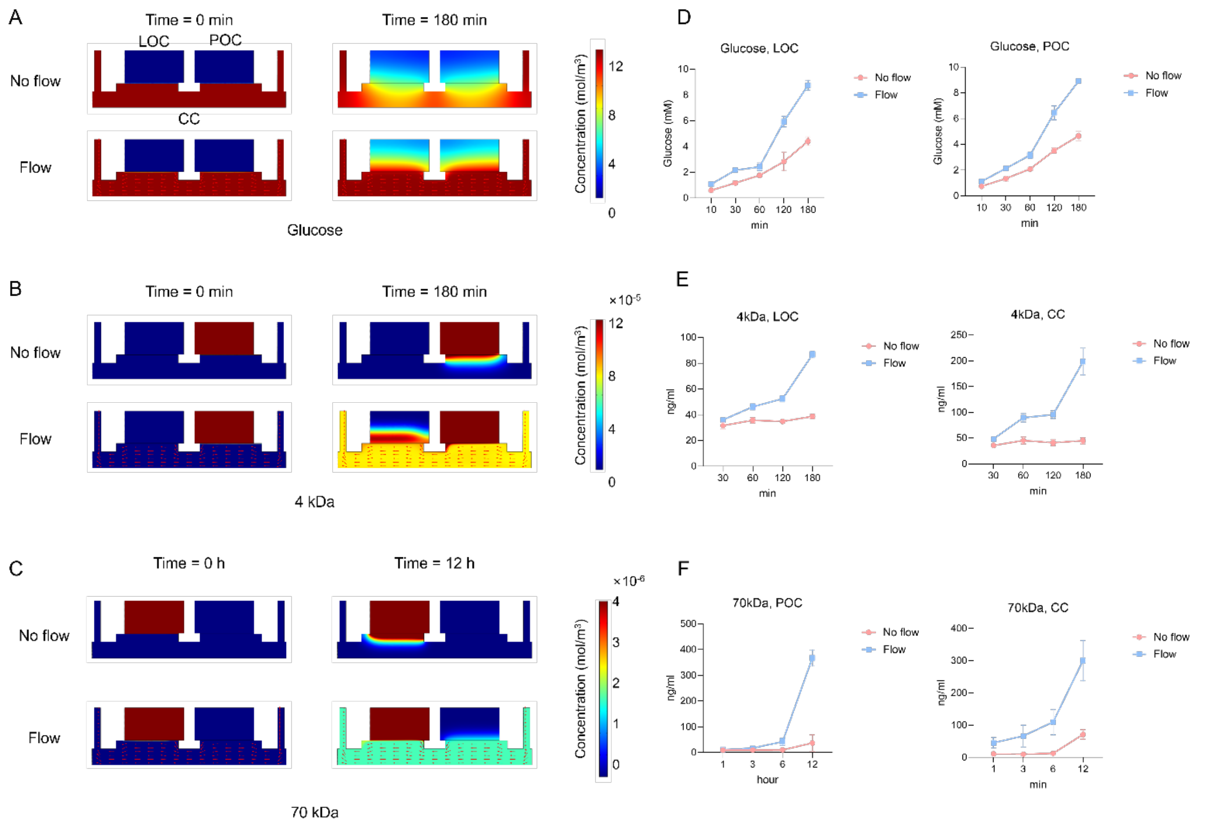

Figure S2. Computational simulation of convective flow and mass transport in the MOD platform. Simulation analysis of glucose, 4 kDa and 70 kDa FITC-dextran concentrations within the device under the influence of fluid flow in the CC. Time-dependent concentration profiles of (A) glucose, (B) 4 kDa FITC-dextran under static (no flow) and flow conditions at 0 and 180 minutes, and (C) 70 kDa FITC-dextran under static and flow conditions at 0 and 12 hours. Corresponding experimental measurements of (D) glucose, (E) 4 kDa FITC-dextran under static (no flow) and flow conditions at 0 and 180 minutes, and (F) 70 kDa FITC-dextran under static and flow conditions at 0 and 12 hours. All data represent mean ± SEM (n = 3).

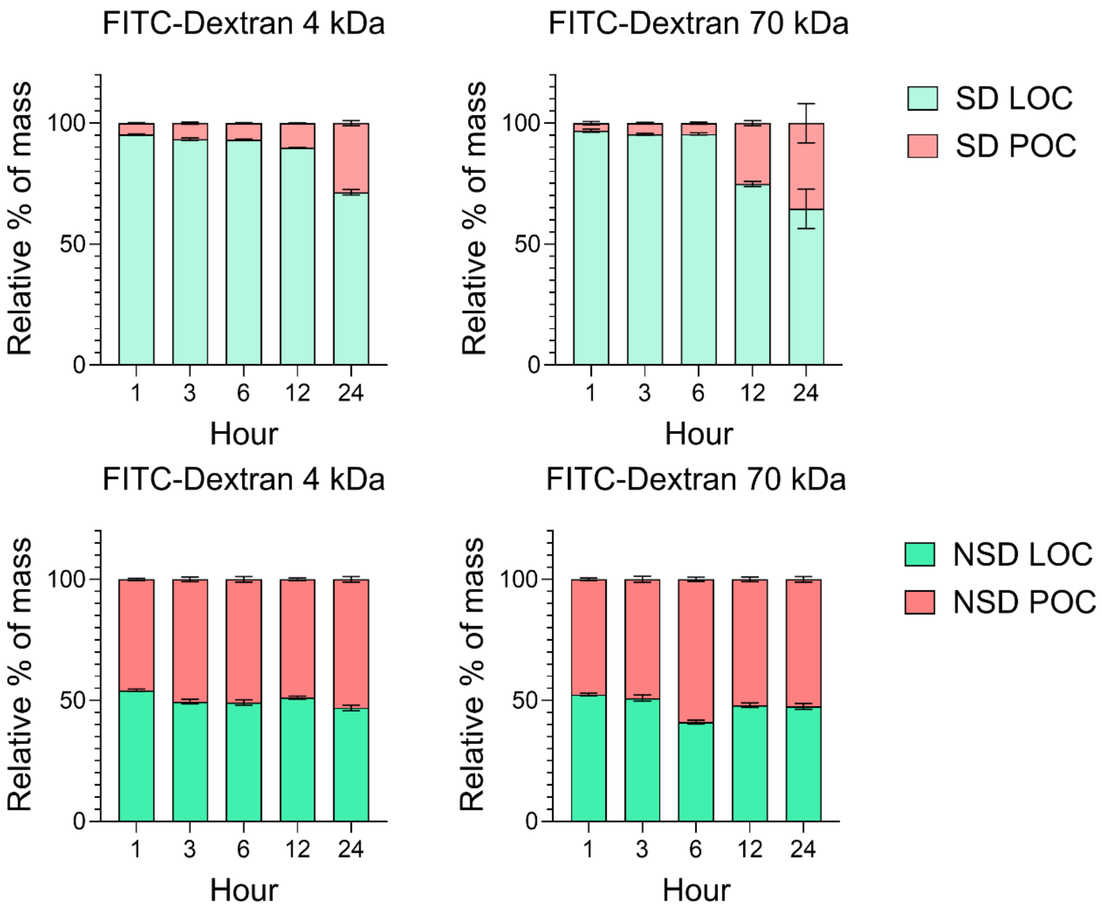

Figure S3. Diffusion dynamics of FITC-dextran in separated vs. non-separated devices. Comparison of mass diffusion between chambers in separated and non-separated devices (n = 4).

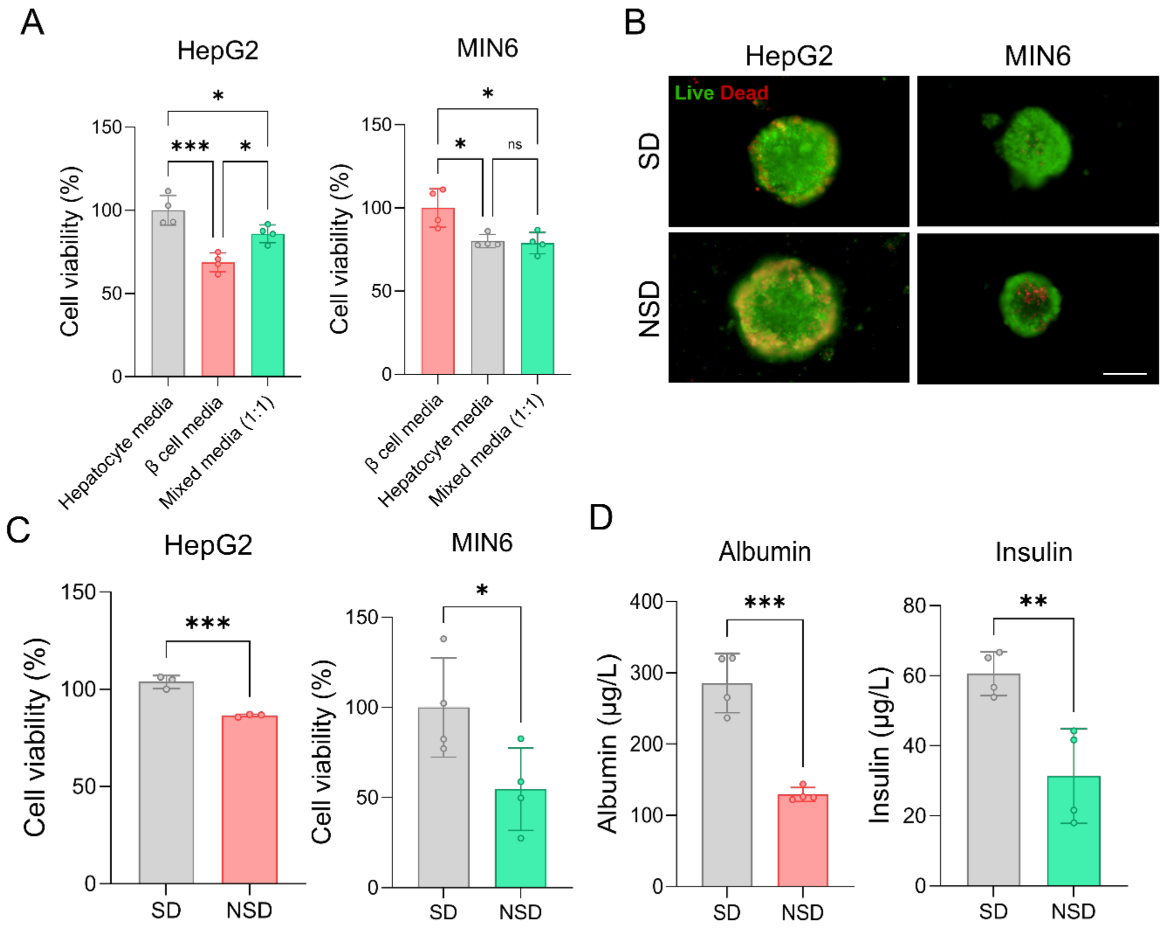

Figure S4. Cell viability and function in a media-separated culture system. (A) Cell viability of HepG2 and MIN6 using alamarBlue assay after 48 hours of culture in well plate (n = 4, **p* < 0.05, ****p* < 0.001). (B) Live/dead staining of HepG2 and MIN6 spheroids after 48 hours of culture in separated device (SD) and non-separated device (NSD) (Scale bar = 100 μm). (C) Cell viability of HepG2 and MIN6 using alamarBlue assay (n = 3-4, **p* < 0.05, ****p* < 0.001 vs. SD). (D) Albumin secretion in HepG2 and insulin secretion in MIN6, based on the separation of culture media, measured by ELISA. (n = 4, ***p* < 0.01 and ****p* < 0.001 vs. SD).

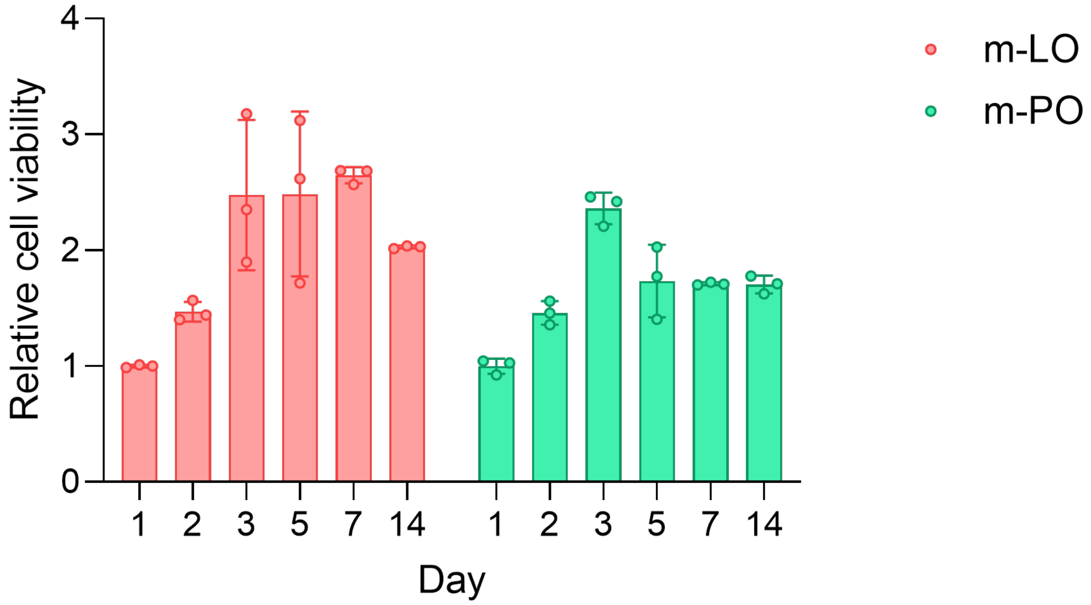

Figure S5. Sustained organoid viability for up to 14 days under SD conditions. Cell viability of m-LO and m-PO cultured for 14 days (n=3)

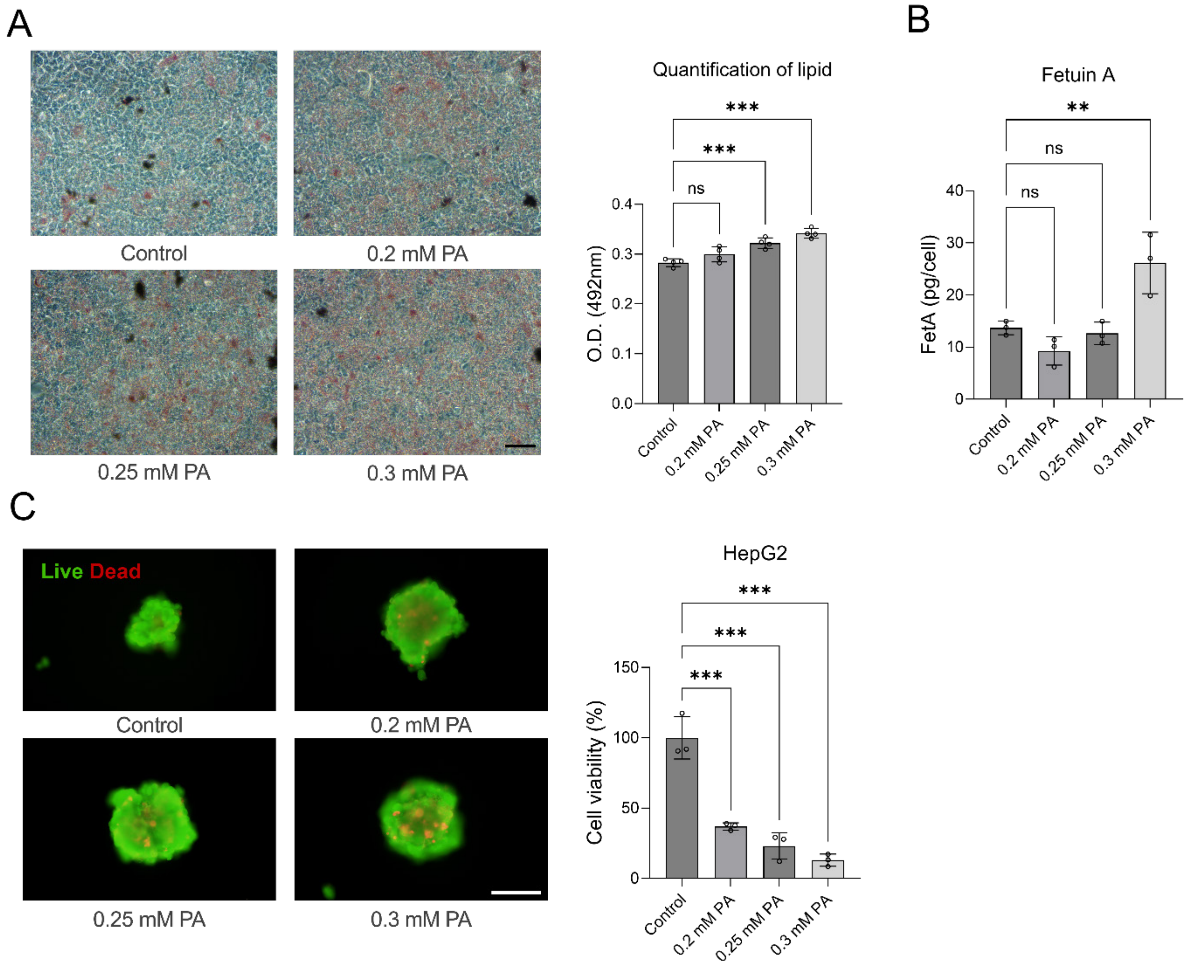

Figure S6. Lipid accumulation, Fetuin A (FetA) release, and viability of HepG2 exposed to high concentration of fatty acids to recapitulate metabolic dysfunction-associated steatotic liver disease (MASLD). (A) Oil Red O staining and quantification of lipid accumulation in HepG2 without (Control), or with PA treatment (0.2 mM, 0.25 mM, 0.3 mM PA) (n = 4, $***p < 0.001$ vs. Control) (Scale bar = 200 μm). (B) FetA secretion in HepG2 measured by ELISA (n = 3, $**p < 0.01$ vs. Control). (C) Live/Dead staining and cell viability of HepG2 spheroid exposed to different PA concentration after 24 hours of PA treatment (n =3, $***p < 0.001$ vs. Control) (Scale bar = 200 μm). Cell viability was assessed using the Live/Dead assay and the alamarBlue assay.

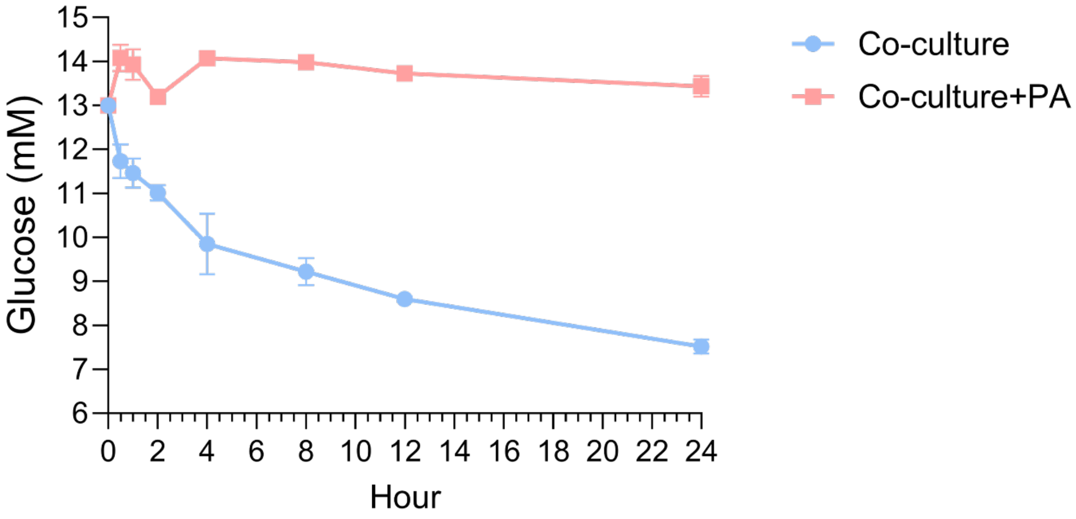

Figure S7. In vitro glucose tolerance test (GTT) using cell lines in the multi-organoid device (MOD). Quantification of glucose level during 24 hours after injection of 13 mM glucose (n = 3).
